# Supplementary material for: Taking stock of national climate policies to evaluate implementation of the Paris Agreement
Source: Nat Commun. 2020 Apr 29;11:2096. doi: 10.1038/s41467-020-15414-6 (PMC7190619; doi:10.1038/s41467-020-15414-6)
Supplement: Supplementary file 1 — Supplementary Information [file 41467_2020_15414_MOESM1_ESM.pdf]

Supplementary Information to ‘Taking stock of national climate policies to evaluate implementation of the Paris Agreement’

Roelfsema et al.

Contents

Supplementary Figures ... 3
Supplementary Tables ... 10
1 Supplementary Note: Scenario protocol for the model comparison ... 46
1.1 Country and region definitions ... 46
1.2 Climate policy database and selection of high impact climate policies ... 46
1.3 Nationally Determined Contributions ... 47
2 Supplementary Note: Kaya indicator framework and uncertainty ... 48
2.1 Kaya indicator framework ... 48
2.2 Uncertainty ... 49
3 Supplementary Note: Assessment of policy impact on GHG emissions in the context of other literature sources ... 50
3.1 Effort sharing ... 50
3.2 National policies and carbon budgets ... 50
4 Supplementary Note: Model documentation and policy implementation ... 51
4.1 Integrated Assessment Model descriptions ... 51
5 Supplementary References ... 75

Supplementary Figures

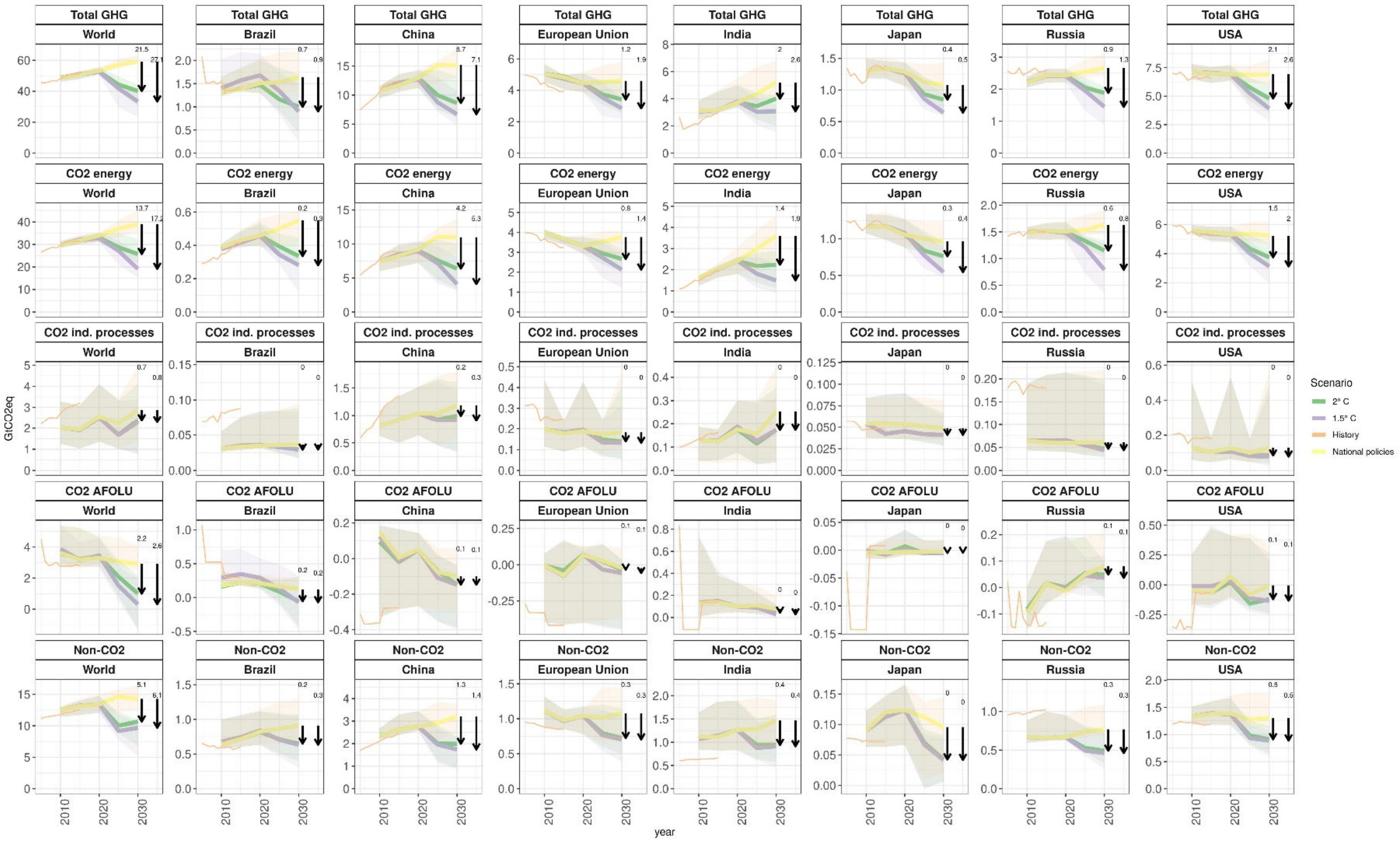

Supplementary Figure 1 Decomposition of total greenhouse gas emissions into CO_2 energy, CO_2 industrial processes, CO_2 Agriculture and other land use (AFOLU) and non-CO_2 emissions. Arrows with number a on top show the emissions gap in $GtCO_2eq$.

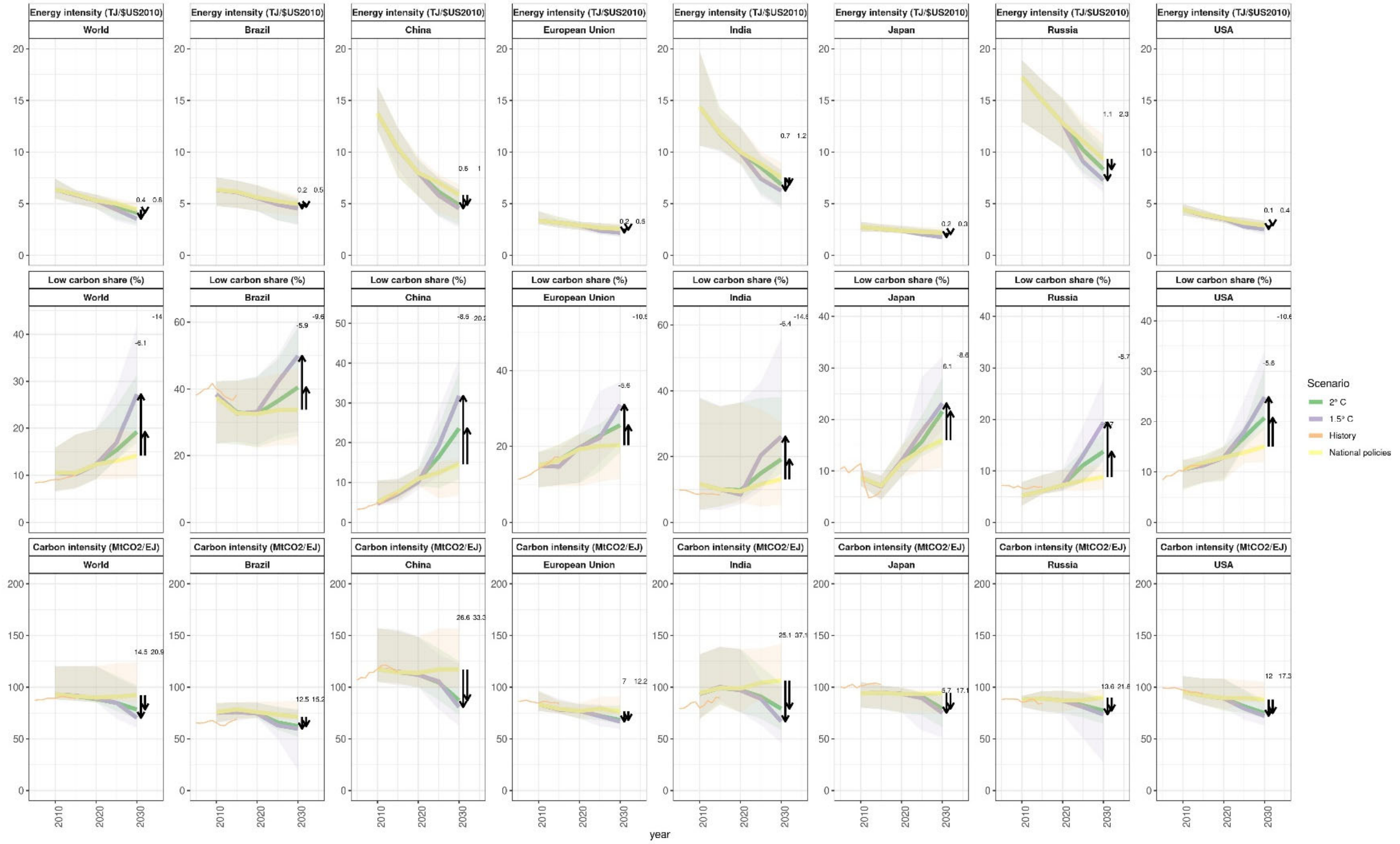

Supplementary Figure 2 Decomposition of CO_2 emissions using Kaya identity into energy intensity (final energy/GDP), low carbon share of final energy and CO_2 intensity of fossil final energy

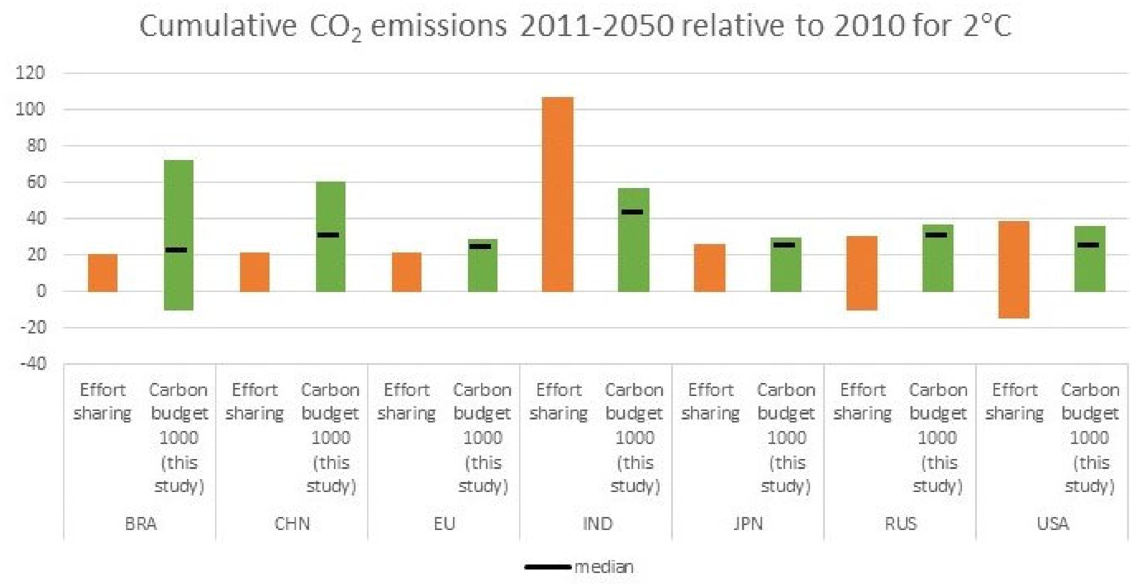

Supplementary Figure 3: Comparison of G20 country emission years (cumulative emissions 2011-2050 relative to 2010) with effort sharing range for carbon budget 1000 GtCO2 from CD-LINKS project. The effort sharing ranges are calculated by only one model, the FAIR model[1]

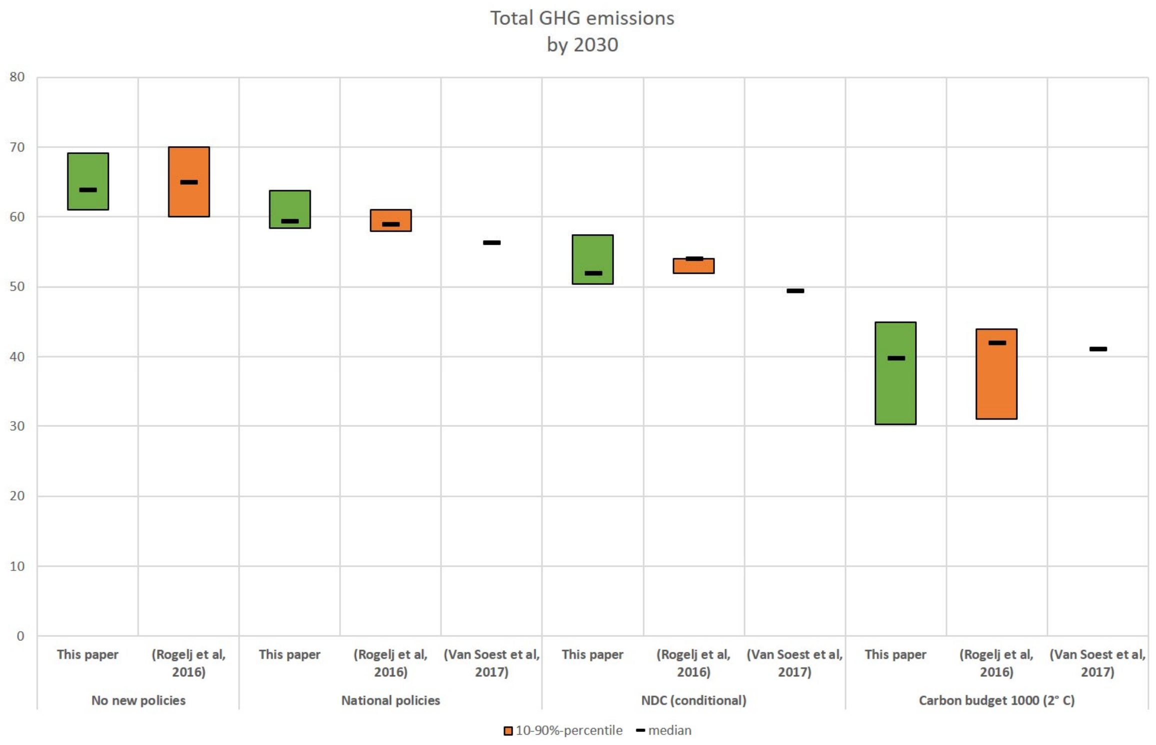

Supplementary Figure 4: Comparison of total GHG emissions on a global level for the No new policies, National policies, NDC (conditional) and Carbon budget 1000 scenario between this study and Rogelj et al[2] and Van Soest et al[3].

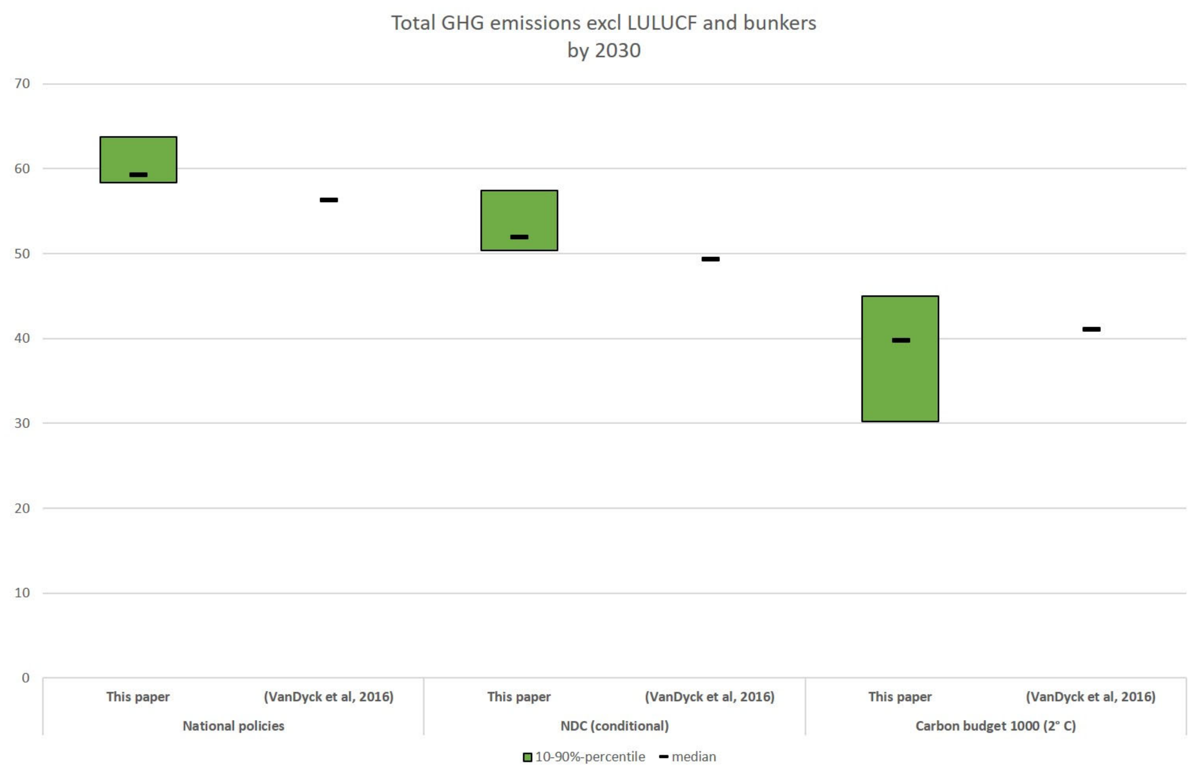

Supplementary Figure 5: Comparison of National policies, NDC and carbon budget 1000 scenario on global level with VanDyck et al[4]. Total emissions exclude LULUCF and bunkers

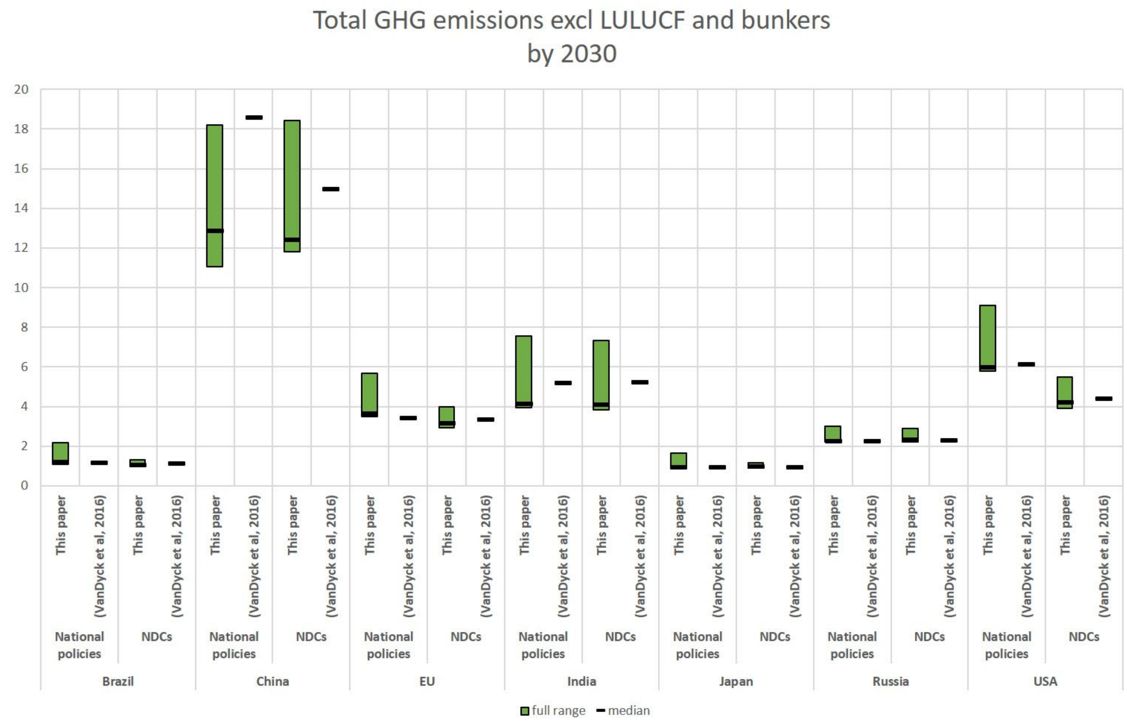

Supplementary Figure 6: Comparison of National policies for seven large G20 countries with VanDyck et al [4]. Total emissions exclude LULUCF and bunkers

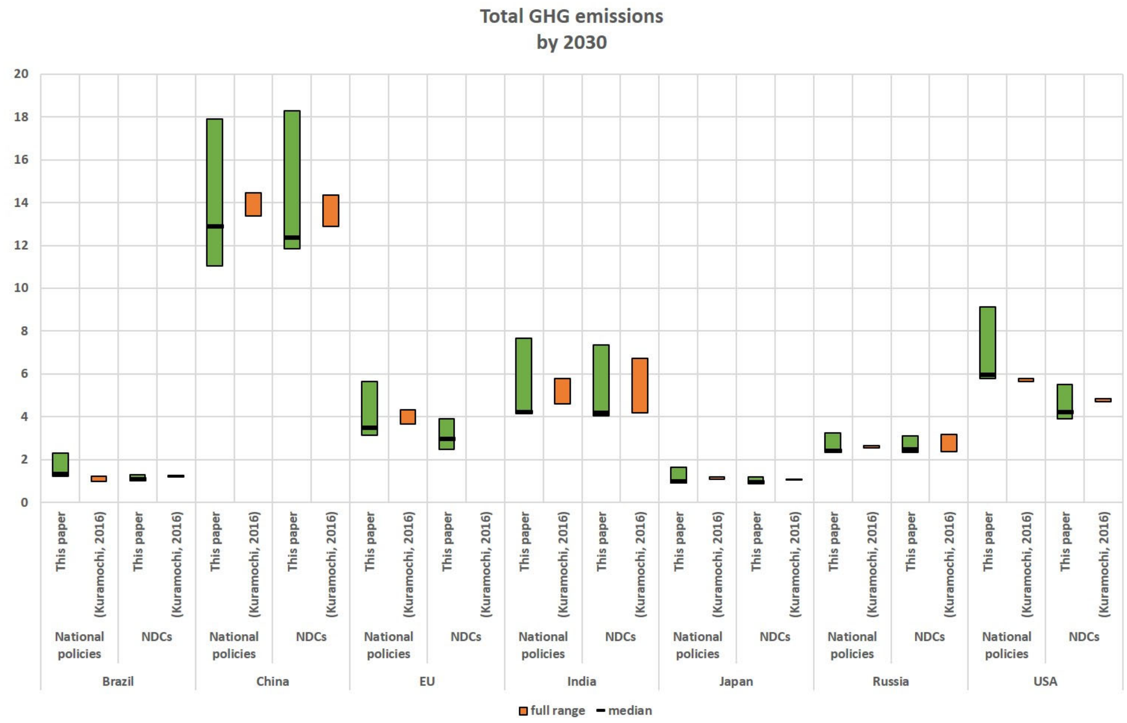

Supplementary Figure 7: Comparison of National policies and NDCs of seven large G20 countries with Kuramochi et al[5]. Total emissions exclude LULUCF and bunkers

Supplementary Tables

Supplementary Table 1 Total GHG emissions in 2010 for G20 countries and countries with implemented climate policies, but not included in assessment

	Country	ISO	Total Kyoto emissions (2010)
	World	EARTH	47,100,000
Included	Argentina	ARG	450,000
	Australia	AUS	554,000
	Brazil	BRA	1,550,000
	Canada	CAN	880,000
	China	CHN	10,600,000
	France	FRA	473,000
	Germany	DEU	932,000
	India	IND	2,140,000
	Indonesia	IDN	2,140,000
	Italy	ITA	474,000
	Japan	JPN	1,150,000
	Mexico	MEX	690,000
	Republic of Korea	KOR	625,000
	Russia	RUS	2,510,000
	Saudi Arabia	SAU	533,000
	South Africa	ZAF	525,000
	Turkey	TUR	357,000
	the United Kingdom	GBR	608,000
	the United States	USA	6,580,000
	European Union	EU28	4,490,000
	G20 countries		35,774,000
	Seven large emitting countries		29,020,000
Not included (with implemented policies)[1]	Bhutan	BTN	(801)
	Chile	CHL	90,600
	Costa Rica	CRI	6,890
	Ethiopia	ETH	135,000
	Gambia	GMB	2,100
	Kazakhstan	KAZ	305,000
	Mororcco	MAR	102,000
	New Zealand	NZL	62,400
	Norway	NOR	33,500
	Peru	PER	174,000
	Philippines	PHL	202,000
	Singapore	SGP	52,700

[1] https://climateactiontracker.org/countries/, retrieved October 2019

Switerzland	CHE	51,100
UAE	ARE	233,000
Ukraine	UKR	394,000
Total with policies, not included		1,843,489
World	EARTH	47,100,000

Supplementary Table 2 Consulted sources for setting up Climate Policy Database

Name	Sectors covered	Countries	Report/Database	Website
Climate Policy Database	All	All	Database	http://climatepolicydatabase.org/
IEA Addressing Climate Change	All, including Adaptation	50 countries including all IEA countries	Database	http://www.iea.org/policiesandmeasures/climatechange/
IEA Global Renewable Energy	Renewables	126 countries including all IEA countries	Database	http://www.iea.org/policiesandmeasures/renewableenergy/
IEA Energy Efficiency	Energy Efficiency - All	66 countries including all IEA countries	Database	http://www.iea.org/policiesandmeasures/energyefficiency/
Climate Action Tracker	All	30 countries	Country Profiles	http://climateactiontracker.org/countries.html
UNFCCC National Communications	All	Worldwide	Country Reports	http://unfccc.int/national_reports/items/1408.php
LSE Global Climate Legislation DB	All	Worldwide	Database	http://www.lse.ac.uk/GranthamInstitute/legislation/the-global-climate-legislation-database/
OECD Fossil Fuel Support	All	OECD countries	Database	http://stats.oecd.org/Index.aspx?DataSetCode=FFS_AUS
Columbia Law School Database	All	Worldwide	Country Profiles	http://web.law.columbia.edu/climate-change/resources/climate-change-laws-world#http://web.law.columbia.edu/climate-cha
INDCs - UNFCCC	All	Worldwide	Country sheets	http://www4.unfccc.int/submissions/indc/Submission%20Pages/submissions.aspx
ECOLEX	All	Worldwide	Database	https://www.ecolex.org/

REN21	RE and EE	Worldwide	Database	Data download: http://www.ren21.net/status-of-renewables/ren21-interactive-map/; Report: http://www.ren21.net/future-of-renewables/global-futures-report/
Kevin Boulder Thesis	Climate Strategies	Worldwide	Database	Excel file provided
Enerdata	Building standards	A few countries	Database	Export excel: https://www.wec-policies.enerdata.eu/world-overview.php#BC-residential
Industrial Efficiency Policy Database (IEPD)	Industrial efficiency	15 countries	Country profiles	http://iepd.iipnetwork.org/
Transport policy	Vehicle and fuel energy and emissions standards	10 countries	Country profiles	Transportpolicy.net
Dieselnet	Emissions standards	A few countries	Country profiles	https://www.dieselnet.com/standards/
REEGLE	RE and EE	All?	Country profiles	http://www.reegle.info/countries/a
RES Legal	Renewables	EU members	Country profiles	http://www.res-legal.eu/
OECD Policy Instruments for the Environment	Fiscal/Financial/Regulatory	OECD and 38 others	Database	http://www2.oecd.org/ecoinst/queries/Default.aspx#
OECD Environmental country data	Not policies!! Indicators.	OECD + others	Database	https://stats.oecd.org/Index.aspx?DataSetCode=EPS
OECD Science, technology and industry outlook		OECD	Country surveys	http://qdd.oecd.org/DATA/STIOb_COUNTRY_ITEM_TOPIC_POLICY_SOURCE/.SVN..STIO_2012?Page=1
Investment and R&D	R&D	All	Country profiles	https://www.innovationpolicyplatform.org/content/statistics-ipp?l=G_XGDP;v3;s;;IND
World Bank INDC data	INDCs	All	Database	http://spappssecext.worldbank.org/sites/indc/Pages/mitigation.aspx

WTO Environmental Database	Trade-relevant env. policies	All	Database	https://www.wto.org/english/tratop_e/envir_e/envdb_e.htm
State incentives of RE & EE	RE and EE	US	State list	http://www.dsireusa.org/
State Energy Efficiency Policy	EE all	US	Database	http://database.aceee.org/
IEA Clean Coal Database	Emissions standards	All		http://www.iea-coal.org.uk/site/2010/database-section/emission-standards?
Industrial Efficiency Programs	Industry	All		http://www.iipnetwork.org/databases/programs
GBPN - Building Policies for a Better World	Buildings	A few EU & US states		http://www.gbpn.org/databases-tools
APEC Energy Standards	Appliances	21 countrie s		http://apec-esis.org/
ICAP Emissions Trading Schemes	Industry (?)	All National and Regional		https://icapcarbonaction.com/ets-map
EU Climate Change Mitigation Policies and Measures	All, including Adaptation	EU		http://www.eea.europa.eu/data-and-maps/data/climate-change-mitigation-policies-and-measures-1
Deutsche Bank Global Climate	All	All	Report	https://www.db.com/cr/en/docs/Global_Policy_Tracker_20120424.pdf

Policy Tracker				
Asia Regional Integration Centre	All	Asia	Database	https://aric.adb.org/climatechange?seltab=3

Supplementary Table 3: Selected number of high impact policies for G20 countries

**Policy type**	Brazil	China	European Union	India	Japan	Russian Federation	United States of America	Other G20 countries	Total
Renewable electricity policies	6	9	0	8	1	4	1	33	62
Other policies	1	0	0	0	0	0	1	2	4
Transport biofuel blending	4	2	0	4	0	0	3	10	23
Forestry policies	4	3	0	2	2	0	0	8	19
F-gas emission reduction policies	0	0	1	0	1	0	0	1	3
Transport fuel tax	0	0	0	0	0	0	0	2	2
Economy-wide policy targets	0	7	6	0	2	1	0	6	22
Renewable policies in demand sectors	2	2	3	0	0	0	0	1	8
Buildings policies	1	0	0	0	0	0	0	0	1
Transport fuel efficiency standards	1	4	2	4	1	0	2	8	22
New power plant standards	0	0	0	1	0	0	1	2	4
Building standards	0	0	0	0	0	0	0	2	2
Existing power plant standards	0	1	0	0	0	0	0	0	1
Industry policies	0	3	0	4	1	0	0	1	9
Building codes	0	1	2	0	1	0	4	2	10
Electric vehicle policies	0	4	0	1	1	0	0	0	6
Carbon taxes, emission trading	0	0	1	0	0	0	0	1	2
Energy tax/subsidies	0	0	0	0	6	0	0	0	6
Fossil-fuel production policies	0	0	0	0	0	2	1	2	5
Other buildings policies	0	0	0	0	0	1	2	0	3
Agricultural policies	0	0	0	0	0	0	1	0	1
	19	36	15	24	16	8	16	81	215

Supplementary Table 4 Documentation of policy implementation in global IAMs

**Model/policy**	**AIM V2.1**	**IMAGE 3.0**	**MESSAGEix-GLOBIOM_1.0**	**POLES CDL**	**REMIND-MAgPIE 1.7-3.0**
**Carbon tax, emission trading**	A carbon tax on region level is the main policy instrument of the model, increasing the cost of fossil energy carriers and increasing the cost-effectiveness of energy savings measures. The carbon tax is endogenously determined by emissions constraints parameters.	A carbon tax on region and sector level is the main policy instrument of the model, increasing the cost of fossil energy carriers and increasing the cost-effectiveness of energy savings measures. The carbon tax is an input parameter of the model. Emissions trading is implemented through imposing a carbon tax on GHG emissions and sectors covered, such that the emission reduction target (or cap on GHG emissions) is met.	*not implemented*	The carbon value is the main mitigation instrument across the entire economy. It is different according to the region and sector. It is an input of the modeller used to reach a given carbon emission budget. No explicit carbon trading; the EU ETS is modelled as a single carbon price for participating countries and sectors.	Regional carbon taxes that apply for all GHG emissions are the main policy instrument in the policy scenarios. In the NDC scenario, they are iteratively adjusted, such that the regional 2025/2030 emissions target is met, with exogeneous assumptions on the temporal profile of the tax. For scenarios with global emissions budgets, similarly the harmonized global tax rate is adjusted iteratively so as to meet the budget.

**Renewable electricity policy**	Renewable share and capacity targets are exogenously input as the share in the total electricity. The capacity targets are translated into the power generation and shares accordingly by assuming capacity factors. To realize these targets, logit parameters are endogenized.	Renewable share: first the share of technologies used for electricity production is determined in the usual way (multinomial logit function, see general description of IMAGE policy implementation). Then, if the annual increase would reach a renewables share lower than the imposed target, the ratio of renewable to fossil technologies is increased until the total renewable share is achieved, keeping the ratio between the renewable technologies, and also between the non-renewable technologies, the same. Renewable capacity targets: these are input parameters of the model, and enforce installation of specific capacities in addition to the outcome of the multinomial logit function in the same way	Renewable share: Share targets in general can be applied for different energy levels. As MESSAGE models 11 global regions, national targets are re-calculated, based on historical data, into regional targets. Should multiple national targets within a model region exist, then these are aggregated. If these multiple national targets within a single model region differ in their definition (e.g. non-fossils as a share of primary energy and renewables as a share of electricity generation), then these are recalculated to a single type of share constraint in order to obtain the aggregated impact within a single region. Renewable capacity targets: renewable capacity targets are recalculated into (powerplant) activity	Renewable capacities are in competition with non-renewable technologies through a multinomial logit function while taking into account the non-dispatchable nature of wind and solar energy sources. The decreasing value of wind and solar with their penetration is included. Feed-in tariffs modify the competitiveness of renewables, by technology. A non-cost factor representing the technological maturity and choice preferences between technologies can be altered to represent regulatory measures, non-cost policies or non-market-related consumer choice.	Short term targets for absolute renewable deployment (in GW) or renewable shares (in %) are implemented in all regions. For the former, country targets are summed up, while the latter is only used for native model regions (i.e. EU28, Japan, USA, China, India)

		as for renewable electricity targets.	which is used as direct lower bound in the model.		

**Renewable policy in demand sectors**	Renewable targets in demand sectors are not implemented.	Renewable targets in demand sectors can only be implemented iteratively by implementation of individual measures (e.g. car renewable electricity targets, efficiency targets, efficiency standards, subsidies of electric cars, biofuel standards and fuel tax in the transport sector) such that the renewable target is achieved. If possible, the policy mix should be based on existing country climate- and energy plans.	Renewable targets in demand sectors Here only biofuel targets or renewable share targets are directly implemented as constraints in the model. As MESSAGE models 11 global regions, national targets are re-calculated, based on historical (2010) data, into regional targets. Should multiple national targets within a model region exist, then these are aggregated. If these multiple national targets within a single model region differ in their definition (e.g. non-fossils as a share of primary energy and renewables as a share of electricity generation), then these are recalculated to a single type of share constraint in order to obtain the aggregated impact within a single region.	Renewables adoption in demand sectors is triggered by the carbon value in these sectors, which will favour low-carbon options in the multinomial logit function.	The share targets for biofuels in transport are implemented on the level of secondary energy liquids, with a lower share to account for non-transport liquids use in buildings and industry.

**Existing power plant standard**	Existing power plant standards are implemented by changing the input coefficient of fuel.	Existing power plant standards can be implemented through changing the efficiency of existing power plants starting after a specific year, which are both input parameters of the electricity model.	Existing power plant standards have not been directly implemented. The model is calibrated to IEA energy production/generation statistics and certain technology transitions are already assumed as part of the overall scenario design, e.g. once unabated coal power plant capacity has reached the end of its life time a shift to newer, cleaner technologies is assumed.	Power plant standards are input parameters for historical plants.	No standards on existing power plants are implemented.
**New power plant standard**	New power plant standards are not explicitly considered.	New power plant standards are implemented by specifying a maximum efficiency or CO2-intensity for new power plants (input parameter), which prevents installation of less efficiency power plants even if this is cost-effective (allocation is done with multinomial logit function).	New power plant standards have not been directly implemented. The model is calibrated to IEA energy production/generation statistics and certain technology transitions are already assumed as part of the overall scenario design, e.g. once unabated coal power plant capacity has reached the end of its life time a shift to newer, cleaner technologies is assumed.	The standards of new power plants can be adjusted by the evolution of efficiency in the future.	In the case of the US, this is implemented by disabling new construction of coal power plants without CCS.

Model/policy	WITCH2016	COPPE-COFFEE 1.0	DNE21+ V.14	GEM-E3
**Carbon tax, emission trading**	Carbon taxes can be implemented globally or regionally, and on a generic sector such energy or land use or on a specific fuel. Similarly, a global or coalition specific emission trading market can be implemented for the energy system, based on a per-specified GHG emission cap.	In mitigation scenarios the model can use a combination of carbon tax and/or carbon budgets. When using carbon budgets, the cost of limiting carbon emissions is the shadow price (dual value). Emissions trading is available in the model, and it is used to achieve the global minimum cost. In the basic form of the model, there is no constraint on emissions traded (regions can trade freely).	A carbon tax on region is an input parameter of the model.	Carbon taxes and the energy system soft linkage with energy system models or IAMs (PRIMES for the EU28 and IMAGE for non-EU regions in these scenarios) are the main enablers of the low-carbon transition in this scenario set-up for GEM-E3. Following the transformation of the energy system via the soft linkage, a carbon tax is applied to all unabated emissions so as to ensure the achievement of the emission target and/or the emissions trajectory. For the NDC scenarios this corresponds to a carbon tax on the economic sectors and GHG gases that are mentioned in the original INDC communication of the Parties to UNFCCC so as to achieve the NDCs in 2025/2030. The carbon tax is endogenous and estimated as the dual variable of the emission constraint. It thus serves so as to abate the remaining emissions and meet the 2025/2030 target in the NDC case or the 2010-2050 emission pathway for the budget

				scenarios. For this application, there is no initial allocation of permits and no emission trading takes place. Nevertheless, GEM-E3 features several structures of emission trading schemes, not used in this analysis. Carbon tax revenues are then recycled back to the economy through either i) a reduction of social security contributions, ii) the reduction of indirect taxation or iii) a lump-sum transfer to the households.

**Renewable electricity policy**	Both renewable shares and capacity targets, can be directly implemented in WITCH constraining the model to meet at least the specified target. The shadow price yields the marginal cost of the policy.	The user defines what technologies are a part of the renewable share or capacity (hydro, wind, solar, geothermal, biomass, etc). Renewable share: shares are generally added by user-defined constraints, for each region, which are composed of inequalities equations in the LP. Renewable capacity targets: there are two ways to implement capacity targets: minimum constraint of absolute capacity (unit: MW) or capacity additions (MW/year).	Renewable share targets in electricity generation by region by time point are represented by additional constraints which total renewable generation (e.g. total renewables including hydro) divided by total power generation is equal to an input parameter of the model. Renewable capacity targets are input parameters of the model. For solar and wind, capacity targets are converted to generation targets by using annual capacity factor by region.	GEM-E3 power supply system features 10 power technologies. The power supply mix is exogenously defined via a one-way soft-linkage with other energy system models (PRIMES for the EU28 and IMAGE for the non-EU regions in this case). We thus follow the power mix and RES targets defined in the implementation of scenarios by IMAGE model. In particular, to achieve the soft link for the power mix, GEM-E3 features a Leontief production function for power supply, whose parameters are set equal to the shares of each technology in the power mix of the energy system model.
**Renewable policy in demand sectors**	Renewable targets in demand sectors can only be implemented in 2 generic sectors (electric and non-electric(except road transport)) and in road transport. Similarly to the electricity targets this can be implemented directly as constraints to the model. The shadow price yields the marginal cost of the policy.	Renewable targets in demand sectors can be achieved in different ways. The main approach is through implementation of individual measures (e.g. electrification targets, efficiency standards, biofuel standards and fuel tax). Nonetheless, user-defined constraint (such as for renewable share) can also be applied.	Renewable targets in demand sectors are represented by additional constraints which total renewable energy consumption (e.g. biofuels in transport) divided by total final energy consumption by sector is equal to an input parameter of the model.	Similarly to the power supply sector, for this analysis, the fuel mix of the GEM-E3 for the energy demand sectors is exogenously taken via a one-way soft-linkage. For this purpose, we adjust the fuel mix of private transportation, the fuel mix of public transport modes, the fuel mix of freight transportation, the fuel mix of households, industry and service sectors, including data for the level of

				electrification and the penetration of biofuels by end-user category.
**Existing power plant standard**	Existing power plant standards can be implemented through changing the efficiency of existing power plants starting after a specific year, which are both input parameters of the electricity model.	Existing power plant standards are modelled by changing the input parameters (such as efficiency) of the set of technologies available in the model.	Existing power plant standards are represented by excluding specific power plant options (e.g. low-efficiency coal) by region by time point which does not meet the standards.	No standards on existing power plants are implemented.
**New power plant standard**	New power plant standards are implemented by specifying the efficiency for new power plants (input parameter).	New power plant standards are implemented by limiting the options of technology expansion of the model, preventing certain technologies (available in the model) to be chosen. When a specific standard is not within the range of the current set of technologies available, new technologies were added to represent the new standard.	New power plant standards are represented by excluding specific power plant options (e.g. low-efficiency coal) by region by time point which does not meet the standards.	No standards on new power plants are implemented, but implictly this is taken into consideration through the exogenous input of power supply mix (please refer to the responses of the IMAGE model)

Model/policy	AIM V2.1	IMAGE 3.0	MESSAGEix-GLOBIOM_1.0	POLES CDL	REMIND-MAgPIE 1.7-3.0
**Transport fuel efficiency standard**	The fuel efficiency of total road energy consumption is an input parameter of the model, which is represented by changing the efficiency parameter. Actual model results are affected by the price effects and therefore we changed the parameter manually roughly meet the corresponding target.	The fuel efficiency of new cars and trucks is an input parameter of the model, which is fixed for the target year, and interpolated between the current and target year. Non-fuel costs of cars are changed accordingly.	The effect of fuel efficiency standards of new cars and trucks was implemented via adjusting the autonomous energy efficiency improvement (AEEI) indicators of the MACRO model (linked to MESSAGE) based on the total final energy savings as estimated by the IMAGE model.	The fuel efficiency of vehicles can be defined as input parameter if the modelling does not otherwise reach the policy objective (via price-induced technical change with fuel prices and carbon prices, and via autonomous technical improvements).	REMIND does not differentiate different vehicle efficiency classes. Therefore, these policies are represented by implementing an upper bound on final energy use in transport, informed by results from the IMAGE modelling.
**Transport biofuel standard**	Transport biofuel targets is assumed to be in the share of biofuel consumption in the total road energy consumption. We change the logit parameter to hit the target share.	Transport biofuel targets consist of either a mandatory minimum volume of biofuels in the total fuel supply, or sets a minimal share of biofuels. As the TIMER model only includes vehicles that drive on one fuel, biofuel blending is modelled by fixing the share of new biofuel cars and fossil fuelled cars in a specific year. This share is an input parameter to the model, and works in the same way as for the renewable	As MESSAGE models 11 global regions, national targets are re-calculated, based on historical (2010) data, into regional targets. Should multiple national targets within a model region exist, then these are aggregated. The constraint is applied to the transport sector.	The share of biofuels used in conventional engines is determined by the relative costs of fuels. An evolving maximum blend is included.	The share targets for biofuels in transport are implemented on the level of secondary energy liquids, with a lower share to account for non-transport liquids use in buildings and industry.

		electricity share (by changing the result of the multinomial logit function).			
**Electric vehicle policy**	Electric vehicle targets, in terms of share of new electric car energy usage in the total road energy consumption, is implemented in the same way as is done for biofuel standards.	Electric vehicle targets, in terms of share of new electric cars in the total fleet, is implemented in the same way as is done for biofuel standards.	Share targets in general can be applied for different energy levels. As MESSAGE models 11 global regions, national targets are re-calculated, based on historical (2010)data, into regional targets. Should multiple national targets within a model region exist, then these are aggregated. The constraint is applied specifically to the transport sector.	EVs develop based on a multinomial logit function (with elasticities) according to the relative total cost of all vehicles, including their fuel costs (which are impacted by the carbon value).	Absolute target for number of electric vehicles in stock are directly implemented as lower bound.

**Building standard**	Building codes (standards) are implemented by changing the energy efficiency parameter in the building sector.	Building codes (standards), in terms of maximum energy use per m2, is implemented by specifying the heating efficiency (MJ/m2/HDD) for the target year, inducing use of more efficient heating technologies and increased insulation. The model interpolates this efficiency between the current and target year. Other services such as cooling and appliances are not targeted (yet).	The effect of building codes (and other efficiency measures in the building sector such as standards for appliances and lighting) was implemented via adjusting the autonomous energy efficiency improvement (AEEI) indicators of the MACRO model (linked to MESSAGE) based on the total final energy savings as estimated by the IMAGE model.	The building stock is defined with low consumption, medium consumption and standard buildings. The development of low and medium consumption buildings is linked to a return on investment taking into account energy prices and technological development (maturity of new technologies).	In this version, building standards are not represented.
**F-gas emission reduction targets**	F-gas emissions reduction policies are not implemented.	F-gas emission reduction targets are implemented by applying a carbon tax only to F-gases such that the required emission level is achieved.	*not implemented*	SF6, PFCs: emissions follow MACCs considering the economy-wide carbon price. HFCs: the Kigali agreement was considered reached (exogenous trajectories of emissions per country/region).	F-Gas emissions are an exogeneous parameter in the scenarios, and different pathways are used for the different policy scenarios.

**Fossil-fuel production policies**	Fossil fuel production intensity targets are not implemented.	Fossil fuel production intensity targets are defined in terms of CO2/CH4 per energy used (GJ). It is assumed that the oil and gas production for each region remains at the same level as in the baseline scenario. As this is an end of pipe measure, additional flaring/venting measures are implemented that decrease GHG emissions to the level that would achieve the annual reduction target of the oil/gas emission intensity.	*not implemented*	The emission factor of CH4 for gas, coal and oil production follow MACCs considering the economy-wide carbon price, on top of the underlying activity evolution (gas, coal, oil production).	Not represented, as emissions intensity of fuels is exogeneous parameter in the model.
**Other**	Fossil fuel taxes are set as polices targeting. Industrial and economy wide energy efficiencies are controlled by changing autonomous energy efficiency parameter.	The model allows for setting other taxes or subsidies in addition to carbon tax, such as oil tax and car subsidies resulting in a different allocation of fossil and non-fossil energy carriers or technologies.	The effect of efficiency measures in the industry sector was implemented via adjusting the autonomous energy efficiency improvement (AEEI) indicators of the MACRO model (linked to MESSAGE) based on the total final energy savings as estimated by the IMAGE model.	Energy taxes or subsidies are kept constant in volume; renewable support mechanisms (e.g. feed-in tariffs) are progressively phased-out.	NA

Model/policy	WITCH2016	COPPE-COFFEE 1.0	DNE21+ V.14	GEM-E3
**Transport fuel efficiency standard**	This can be done only for road transport of passenger and freight vehicles. Similarly to the electricity targets, it can be specified directly as a constrain in the model. The shadow price yields the marginal cost of the policy.	The fuel efficiency of new vehicles (cars, busses, trucks, etc) is an input parameter of the model. COFFEE uses the same approach as for new power plant standard: by limiting the available options of fleet expansion (through sales of new vehicles) of the available range of technologies (cars, busses, trucks, etc). Therefore, an specific target is met by allowing a combination of vehicles.	The fuel efficiency of new cars and trucks is represented by excluding specific vehicle options (e.g. small low-efficiency internal combustion engine passenger vehicle) by region by time point which does not meet the standard.	The fuel efficiency of new cars and trucks is an input parameter of the model, calibrated to detailed energy system models
**Transport biofuel standard**	This can be done only for road transport of passenger and freight vehicles. Similarly to the electricity targets, it can be specified directly as a constrain in the model. The shadow price yields the marginal cost of the policy.	Transport biofuel targets are modelled by the combination of three approaches: i) the model has the options of blending biofuels with fossil fuels up to a given range (e.g. from 0% to 50%); ii) there are several technology options for producing advanced biofuels, which replaces conventional fuels (diesel, gasoline, kerosene and bunker); iii) There are vehicles options that can use blended biofuels, conventional or advanced fuels. There are also a	Transport biofuel targets are represented by additional constraints which total biofuel consumption divided by total final energy consumption in transport sector by region by time point is equal to an input parameter of the model.	See column D. Biofuel shares are specified through the one-way soft-link with energy system models (IMAGE)

		few options of flex-fuel vehicles (e.g. gasoline/ethanol).		
**Electric vehicle policy**	This can be done only for road transport of passenger and freight vehicles. Similarly to the electricity targets, it can be specified directly as a constrain in the model. The shadow price yields the marginal cost of the policy.	Electric vehicle targets can be achieved the same way as renewable capacity targets: by share of the fleet or through share of sales of new vehicles.	Electric vehicle target, in terms of the number of electric vehicles is an input parameter of the model.	See column D. Electric vehicles shares are specified through the one-way soft-link with energy system models (IMAGE), in particular apart from the fuel mix for passenger transport, we also adjust the share of new electric, plug-in-hybrid and conventional vehicles taking stock of the input from PRIMES model and other available input from IAM models and adjusting accordingly for non-EU regions.

**Building standard**	Currently the model cannot represent the building sector.	Building codes (standards) are simplified in COFFEE. There assumptions of parameters (heating and cooling efficiency) for the determination of the specific demands of the residential sector, which are not completely endogenous at this time. The model has limited options of energy efficiency for all energy services included in the model.	Building standard, in terms of energy savings in building sector is represented by additional constraints which total final energy consumption in building sector in policy scenarios is smaller than that in baseline by specific amount that is an input parameter of the model.	Not represented
**F-gas emission reduction targets**	F-gas emission reduction targets are implemented by applying a carbon tax only to F-gases such that the required emission level is achieved or by applying a generic carbon tax.	The model does not include F-gas at this time, therefore there are no mitigation options.		F-gases are mitigated through the imposition of the carbon tax. GEM-E3 features a MAC curve for non-CO2 GHGs which has been estimated from input taken by the GAINS model.
**Fossil-fuel production policies**		Fossil fuel production intensity targets are defined by adjusting the use of mitigation options for the oil and gas sector. For instance, there flaring, venting and gas recuperating options for each region and type of oil/gas reservoir (e.g. onshore and offshore). There are also options of recuperating methane in some coal reservoirs. Energy efficiency options for fossil fuel production are not included at this time.		Fossil fuel production intensity targets are remain the same as in the baseline scenario. MAC curves for CH4 emissions imply end of pipe abatement measures for the scenarios, depending on the carbon tax level.

**Other**	The model allows for setting other taxes or subsidies and coalition emission trading markets in addition to carbon tax, such as oil tax and car subsidies resulting in a different allocation of fossil and non-fossil energy carriers or technologies.		CO2 intensity targets (CO2/TPES), energy intensity targets (TPES/GDP), energy consumption targets (TPES and total energy consumption in industry sector relative to those in baseline), primary energy consumption and coal consumption targets (cap), and gas and oil import targets (share) are represented by additional constraints which are input parameters of the model.	

**Model/policy**	***AIM/Enduse[Japan]**	***BLUES**
Carbon taxes, emission trading	Carbon tax trajectory (based on the national carbon budget in each scenario)	The model takes either carbon pricing or an emissions budget. No emissions trading implemented.
Renewable electricity targets	Renewable capacity targets (Calculated from generation share target according to the NDC: 22% in 2030) by 2020 and 2030 in the NPi and NDC scenarios, respectively.	Shares of renewable sources in power generation are implemented via constraints on activity, capacity or both.
Renewable targets in demand sectors	N/A	Transport biofuel targets are modelled by the combination of three approaches: i) the model has the options of blending biofuels with fossil fuels up to a given range (e.g. from 0% to 50%); ii) there are several technology options for producing advanced biofuels, which replaces conventional fuels (diesel, gasoline, kerosene and bunker); iii) There are vehicles options that can use blended biofuels, conventional or advanced fuels. There are also a few options of flex-fuel vehicles (e.g. gasoline/ethanol).<br>Agriculture sector technological options include solar and biomass driers, biofuel machines.<br>Industrial options also include fue switching through technologies delivering the same end service but utilizing renewable sources.<br>The fuel efficiency of new vehicles (cars, busses, trucks, etc) is an input parameter of the model. COPPE-MSB uses the same approach as for new power plant starndard: by limiting the available options of fleet expansion (through sales of new vehicles) of the available range of technologies (cars, busses, trucks, etc). Therefore, a specific target is met by allowing a combination of vehicles.

Existing power plant standards	N/A	Existing power plant standards are fixed to current values. These plants can be replaced by new ones with higher efficiency or, in some cases, refurbished to either extend their lifetime (hydro_repot) or improved their efficiencies (bagasse-fired boilers in the sugarcane sector).
New power plant standards	N/A	New power plants have better standards than vintage ones, but their efficiencies do not improve over time.

**Model/policy**	***AIM/Enduse[Japan]**	***BLUES**
Transport fuel efficiency standards	National fuel economy standards	The fuel efficiency of new vehicles (cars, busses, trucks, etc) is an input parameter of the model. BLUES uses the same approach as for new power plant standard: by limiting the available options of fleet expansion (through sales of new vehicles) of the available range of technologies (cars, busses, trucks, etc). Therefore, a specific target is met by allowing a combination of vehicles.
Transport biofuel targets	N/A	Transport biofuel targets are modelled by the combination of three approaches: i) the model has the options of blending biofuels with fossil fuels up to a given range (e.g. from 0% to 50%); ii) there are several technology options for producing advanced biofuels, which replaces conventional fuels (diesel, gasoline, kerosene and bunker); iii) There are vehicles options that can use blended biofuels, conventional or advanced fuels. There are also a few options of flex-fuel vehicles (e.g. gasoline/ethanol).
Electric vehicle targets	N/A	Electric vehicle targets can be achieved the same way as renewable capacity targets: by share of the fleet or through share of sales of new vehicles.

Building Standards	Building energy standards for new constructions (the 1999 standard for residential and commercial buildings)	Building codes (standards) are not explicitly modelled in BLUES. However, appliances sued in buildings can be chosen from a diverse portfolio of options including CFLs and LEDs for lighting, high efficiency appliances for cooling, PV and solar water heating. There are assumptions of parameters (heating and cooling efficiency) for the determination of the specific demands of the residential and commercial sectors, which are not completely endogenous at this time.
F-gas emission reduction targets	N/A	The model does not include F-gas at this time, therefore there are no mitigation options.
Fossil-fuel production intensity targets	N/A	Land use and agriculture are explicitly model at technology level, with various options in land use conversion, crop and livestock production technologies. Intensification of crop and livestock production is explicitly modelled. Mitigation measures such as nitrification inhibitors are not modeled in the version used in this project but has been implemented in a new version being calibrated and tested currently.
Other	Nuclear capacity targets (Lifetime extension to 60 years and new construction based on the NDC)	

Supplementary Table 5 Overview of policy implementation per integrated assessment model

	%-of policies implemented (in 7 large countries)		Reduction relative to No new policies scenario
	# policies	% impact of IMAGE reductions	
**IMAGE 3.0**	94%	100%	-3.8%
**DNE21+ V.14**	64%	81%	-11.2%
**WITCH2016**	62%	63%	-3.8%
**REMIND-MAgPIE 1.7-3.0**	42%	71%	-4.0%
**MESSAGEix-GLOBIOM_1.0**	43%	81%	-5.7%
**POLES CDL**	49%	56%	-3.0%
**COPPE-COFFEE 1.0**	51%	50%	-0.6%
**AIM V2.1**	55%	64%	-4.6%
**GEM-E3**	40%	58%	NA

Supplementary Table 6: NDC targets for G20 economies

Party (target year)	Base Year emissions (incl LULUCF)	Base Year emissions (excl LULUCF)	LULUCF emissions Target Year	LULUCF credits	Emissions "conditional" vs 2010 (incl LULUCF)	Emissions "conditional" vs 2010 (excl LULUCF)	Emissions "conditional" at target year (incl LULUCF)	Emissions "conditional" at target year (excl LULUCF)
**EU (2030)**	5,368	5,626	-283		68%	69%	3,093	3,376
**Canada (2030)**	789	736	-28	-30	56%	74%	517	545
**Mexico (2030)**	973	x	0		85%	87%	623	623
**USA (2025)**	6,223	7,228	-970		68%	79%	4,543	5,513
**Argentina (2030)**	670	x	115		110%	107%	469	354
**Brazil (2030)**	2,100	x	0		80%	117%	1,300	1,300
**Australia (2030)**	548	523	34		70%	64%	400	367
**Japan (2030)**		1,408	-76	-37	89%	85%	1,003	1,079
**Korea (Republic) (2030)**		851				84%		536

**China (2030)**		5,976	-250					
**India (2030)**		1,433	-325					
**Indonesia (2030)**	2,881	1,918	439		79%	148%	1,700	1,261
**Russian Federation (2030)**	3,532	3,368	-468	-468	106%	119%	2,357	2,826
**Saudi Arabia (2030)**	1,160	1,160				162%		1,030
**Turkey (2030)**	1,175	1,230	59		254%	207%	928	870
**South Africa (2030)**			-26		97%	103%	506	532

Supplementary Table 7: NDC policies in CD-LINKS protocol

Party	Target year	Policy (includes only countries >0.15 of global 2010 emissions)
China	2030	20% non-fossil fuels in primary energy consumption
	2030	increase the forest stock volume by around 4.5 billion cubic meters on the 2005 level
India	2030	40% cumulative electric power installed capacity from non-fossil fuel based energy sources
	2030	create additional carbon sink of 2.5 to 3 billion tCO_2eq through additional forest and tree cover

Supplementary Table 8: Sources for NDC LULUCF CO2 emissions

**Country**	**Source for LULUCF 2030 projections target year**	**Assumptions for LULUCF 2030 projections**	**Source for LULUCF credits**
EU	Den Elzen et al (2016)[6]		We assume the EU has zero LULUCF credits in 2030
Canada	Den Elzen et al (2016)[6]		(Grassi, Dentener, 2015)[7]
Mexico	Den Elzen et al (2016)[6]	Estimate from Fifth National Communication is used (Den Elzen et al. include range)	
USA	(Grassi, Dentener, 2015) [7]		
Argentina	UNFCCC[8]	Assumption: 2010 emissions are kept constant until 2030	
Brazil	(Grassi, Dentener, 2015) [7]		
Australia	Den Elzen et al (2016)[6]		
Japan	Den Elzen et al (2016) [6]		INDC Japan
Republic of Korea			
China	(Grassi, Dentener, 2015) [7]		
India	(Grassi, Dentener, 2015) [7]		
Indonesia	UNFCCC[9]	National baseline from BAPPENAS presentation (Government of Indonesia, 2015)	
Russian Federation	Den Elzen et al (2016) [6]		(Grassi, Dentener, 2015) [7]
Saudi Arabia		It is assumed that the INDC is excluding LULUCF	

Turkey	UNFCCC[10]	No LULUCF credits estimates available, so we assume full accounting. For this 2013 emissions from BUR are kept constant	
South Africa	UNFCCC[11]	Assumption: 2010 emissions are kept constant until 2030	

Supplementary Table 9 Individual impact of most effective policies based on IMAGE model calculations and an overview whether these were implemented in the other eight participating models (1=implemented, 0=not implemented)

Country	Policy target type	GHG Reductions (MT CO_2eq)	IMAGE 3.0	DNE21+ V.14	WITCH 2016	REMIND-MAgPIE 1.7-3.0	MESSAGEix-GLOBIOM_ 1.0	POLES CDL	COPPE-COFFEE 1.0	AIM V2.1	GEM-E3
China	Renewable electricity	409	1	1	1	1	1	1	1	1	1
USA	Power plant standard	366	1	1	0	1	1	0	1	0	1
USA	Fuel efficiency standard cars	242	1	1	1	1	1	1	0	1	0
EU	Building standard	218	1	0	0	0	0	0	0	0	0
EU	Emissions trading	195	1	1	1	1	1	1	0	1	1
China	Fuel efficiency standard cars	160	1	1	1	1	1	1	1	1	0
USA	Flaring and venting regulation	103	1	0	0	0	0	0	0	0	0
EU	Fuel efficiency standard cars	96	1	1	1	0	1	0	0	1	1
India	Fuel efficiency standard cars	90	1	1	1	1	1	1	0	1	0
India	Energy efficiency policy	76	1	1	0	0	1	0	0	1	0
Japan	F-gas policy	64	1	0	1	0	0	0	0	0	1
India	Renewable electricity	37	1	1	1	1	1	1	1	1	1
Brazil	Fuel efficiency standard cars	29	1	1	0	0	1	1	1	1	0

Country	Policy target type	GHG Reductions (MT CO_2eq)	IMAGE 3.0	DNE21+ V.14	WITCH 2016	REMIND-MAgPIE 1.7-3.0	MESSAGEix-GLOBIOM_ 1.0	POLES CDL	COPPE-COFFEE 1.0	AIM V2.1	GEM-E3
Brazil	Biofuel mandate	23	1	1	1	0	1	1	1	1	1
India	Biofuel mandate	20	1	0	1	0	1	0	1	0	1
Japan	Renewable electricity	18	1	1	1	1	0	1	1	1	1
Brazil	Renewable electricity	11	1	1	1	1	1	1	1	1	1
USA	Biofuel mandate	5	1	1	1	0	1	1	1	1	1

Supplementary Table 10 Online model documentation

Model	Coverage IAM model	Documentation
AIM V2.1	Global	http://www-iam.nies.go.jp/aim/data_tools/enduse_model/aim_enduse_manual.pdf
COPPE-COFFEE 1.0	Global/national	https://www.iamcdocumentation.eu/index.php/Model_Documentation_-_COFFEE-TEA (under review)
DNE21+ V.14	Global/national	https://www.rite.or.jp/system/en/global-warming-ouyou/modeltodata/overviewdne21/
GEM-E3	Global/national	https://ec.europa.eu/jrc/en/gem-e3
IMAGE 3.0	Global	https://models.pbl.nl/image/index.php/Welcome_to_IMAGE_3.0_Documentation (including visualization tool)
MESSAGEix-GLOBIOM_1.0	Global	https://message.iiasa.ac.at/en/stable/ (including installation version)
POLES CDL	Global	https://ec.europa.eu/jrc/en/poles
REMIND-MAgPIE 1.7-3.0	Global	REMIND: https://www.pik-potsdam.de/research/transformation-pathways/models/remind<br>MAGPIE : https://www.pik-potsdam.de/research/projects/activities/land-use-modelling/magpie<br>(both including source code)
WITCH2016	Global	https://doc.witchmodel.org/

1 Supplementary Note: Scenario protocol for the model comparison

All scenarios and input parameters used in our analysis are described in the global modelling protocol[12] and accompanying list of high impact policies and policy indicators. A summary of most important assumptions and a short description of scenarios is given in this section. The following scenarios have been used

- No new policies scenario
- National policies scenario
- NDC scenario
- 2 °C scenario
- 1.5 °C scenario

1.1 Country and region definitions

The analysis described here addresses the impact of climate policies of the G20 economies Brazil, China, European Union, India, Russia and the USA. However, the results for Argentina, Australia, Canada, Indonesia, Mexico, Republic of Korea, Saudi Arabia, and South Africa are based on explicit policies also, but were only included in the global results. The Rest of the World (RoW) region that is presented in the paper includes all remaining G20 economies, except for the seven G20 countries that were explicitly addressed. The G20 countries cover approximately 75% of global GHG emissions, and countries with implemented climate policies (according to Climate Tracker) represent approximately 5%.

1.2 Climate policy database and selection of high impact climate policies

To inform the Integrated Assessment Models, a climate policy inventory was developed for the G20 countries[2]. The consulted sources for this were country NDCs that often include a description of policies that are being implemented to meet the NDC reduction targets, literature, national experts and existing policy databases (see Supplementary Table 2). Based on this database, a selection of high impact policies was made, which were secured in the CD-LINKS protocol, and can be found in the Supplementary spreadsheet[13]. Supplementary Table 3 categorises the policies into different policy types. A selection of around ten high impact policies for each G20 country was made with the help of national climate policy experts participating in the CD-LINKS project, but also from outside the project (see worksheet 'high impact policies'). To replicate the impact on GHG emissions, energy and land use, the policies were translated into policy indicators that can be implemented in integrated assessment models (see worksheet 'protocol reference (numerical)'), which is described in the Methods section. The tables in this worksheet show the policy indicators for G20 countries for each sector: economy-wide, energy supply, transport, buildings, industry and AFOLU. These policy targets are classified as ' target, 'alternate interpretation' or 'planned'. The 'target' policies are included in the national policies scenario (NPi), while the planned policies scenario (NPip in protocol) also includes those classified as 'planned'. The latter was not assessed in this report. The 'alternate interpretation' can be used as alternative to the 'target' if this better connects with the model structure.

The spreadsheet also includes NDC emission reduction targets for G20 countries, and many other countries (worksheet 'NDC emission targets'). Note, that some NDCs include additional policy targets besides emission reduction targets (e.g. non-fossil target) (worksheet 'NDC policies'). The NDC

[2] http://climatepolicydatabase.org/index.php/CDlinks_policy_inventory

information is based on the protocol used in the ADVANCE[14,15] project, but was updated with additional information and guidance.

1.3 Nationally Determined Contributions

The Nationally Determined Contributions (NDCs) were included in the NDC. This scenario starts from the National policies scenario, and additionally implements individual G20 country NDC targets (see Table 5), but also NDCs from other countries. In general, these additional emission reductions above national domestic policies were implemented with a carbon tax, resulting in cost-optimal implementation. In addition, China and India also specified renewable energy and forestry targets (see Table 6). Sources for the assumptions on AFOLU CO_2 (i.e. LULUCF CO_2) are specified in Table 7.Supplementary Note: Kaya indicator framework and uncertainty

2 Supplementary Note: Kaya indicator framework and uncertainty

2.1 Kaya indicator framework

Based on the Kaya identity (Equation 1.1) we have analysed the future estimated progress of national climate policies towards the Paris goals of limiting temperature increase relative to pre-industrial levels to well below 2° C by. This was done by designing pathways that keep cumulative emissions between 2011 and 2100 within 1,000 $GtCO_2$ (below 2° C pathway, with high probability) and within 400 $GtCO_2$ (below 1.5° C pathway, with high probability).

$$CO_2 = POP * \frac{GDP}{POP} * \frac{CO_2}{GDP} \quad (1.1)$$

$$\frac{CO_2}{GDP} = \frac{TPES}{GDP} * \frac{CO_2}{TPES} = \frac{TPES}{GDP} * \frac{FE}{TPES} * \frac{CO_2}{FE} \quad (1.2)$$

$$\frac{CO_2}{GDP} = \frac{TPES}{GDP} * \frac{FE}{TPES} * \frac{FE_{fossil}}{FE} * \frac{CO_2}{FE_{fossil}} = \frac{TPES}{GDP} * \frac{FE}{TPES} * \left(1 - \frac{FE_{non-fossil}}{FE}\right) * \frac{CO_2}{FE_{fossil}} \quad (1.3)$$

where

POP = Population
GDP = Gross domestic product
FE = Final energy

The Kaya indicators can support tracking progress of climate policies, both at a global level and for individual countries. At the same time they give guidance on the efforts necessary to enhance ambition in terms of increasing the share of renewable energy technologies and extending the range of efficiency measures. The Kaya identity component CO_2/GDP can be rewritten (see Equation 1.3) to include final energy intensity (FE/TPES), non-fossil share ($FE_{non-fossil}$/FE) and CO_2 intensity of fossil fuels (CO_2/FE_{fossil}) which were used in the analysis.

Instead of looking at CO_2 emissions only, we considered total GHG emissions and broke this down into CO_2-energy, CO_2-industrial processes, CO_2-AFOLU and non-CO_2 GHG emissions (see Figure 1). It shows that CO_2-energy gives a similar picture as total GHG (Kyoto) emissions, except for Brazil. For this reason we have chosen to use total GHG emissions as indicator in our analysis. For the other indicators from Equation 1.3, we have used GDP in terms of Market Exchange Rates (MER). Non-fossil energy in ours assessment includes renewable resources such as solar, wind and biomass, but also nuclear power and electricity generated with fossil fuels together with carbon capture and storage. Total primary energy (TPES) was not included in this analysis as the level depends on the primary energy accounting method used, which differs between models[16]. Figure 2 and Table 1 show that the gap in terms of low carbon share is high for all countries, and differences in energy intensity between countries are most pronounced. These two indicators were used in our analysis.

2.2 Uncertainty

One of the main results of our study is the range of GHG emissions by 2030 representing the impact of climate policy implementation. The uncertainty was decomposed into four drivers: 1) historical calibration, 2) socio-economic growth assumptions, 3) policy impact on GHG emissions and 4) real uncertainty (see Figure 5 in Methods section), and is described in this section. Although this covers most uncertainty, unknown uncertainty due to limited knowledge[17] is not represented as this is difficult to quantify.

The impact of national policies is shown by comparing the national policies scenario with scenarios that have not included new climate policies after 2010. These scenario comparisons were made with nine integrated assessment models (see sections 4 and **Error! Reference source not found.** for more details).

Historical emissions are uncertain as they are in general not directly measured, but estimated based on other indicators (e.g. fuel use in transport). In our analysis, the difference between model emissions and the PRIMAP [18] dataset (version 1.2) is used to give an indication. In addition, the models ensemble represents uncertainty in socio-economic growth rates, as they differ in socio-economic assumptions on GDP, population and energy demand. This uncertainty is represented by the range of GHG emissions in the No new policies scenario.

Then, the uncertainty of policy impact is given by the range of emission reductions between the No new policies scenario and the national policies scenario. This range is the result of the uncertain impact of policies, but also partly due to some models not being able to implement all high impact policies. Table 1 shows that between 42% and 94% of all policies have been implemented by the models. But this does not say much about the impact on GHG emissions. Therefore, we have made an order of magnitude estimate of the impact on GHG emissions of policies not covered by specific models. This was done by first calculating the individual impact of each policy with the IMAGE model (not accounting for overlap between policies), as this model was able to implement most of the high impact policies[3]. Based on the overview of policies that were implemented by each model (see Table 2), we calculated the emissions reductions covered by each model in terms of IMAGE emission reductions. Of course the impact would differ for different models, but this gives the best available order of magnitude estimate of policy impact. The result is an estimate of emissions reductions covered by each model, which is between 50% and 100% (see Table 1), which is equal to 0.4 and 1.3 $GtCO_2eq$. These estimates were used in Figure 5 of the Methods section, that also includes uncertainty ranges for historical calibration, emission growth in the no new policies scenario, policy impact and real uncertainties. The latter uncertainty is represented by the difference in structural form, representing for example different technological learning, and behaviour on price signals or regulation.

[3] Except the building standard for China, medium-trucks efficiency standard in the USA, Electric vehicle production goals for China

3 Supplementary Note: Assessment of policy impact on GHG emissions in the context of other literature sources

3.1 Effort sharing

The cost-optimal budget scenarios from our study were compared to effort sharing ranges based on Van den Berg et al[19] (see Supplementary Figure 3). We have used the results from this paper using the following effort sharing approaches (default settings)

- Ability to pay
- Equal cumulative per capita emissions
- Per capita convergence
- Immediate per capita convergence
- Grandfathering

For most countries, the median of the cost optimal carbon budgets for the period 2011-2050 is close to, but above the maximum of the effort sharing ranges, except for India and the USA. The Indian cost-optimal budget is on the lower side of the effort sharing range, while the median of the US cost-optimal range falls on the higher side of the effort sharing range. Note, that the picture for the period 2011-2100 could be different.

3.2 National policies and carbon budgets

The scenario results on a global and G20 economy level for the national policies, NDCs and 2 °C scenarios were compared with literature outcomes from

- Rogelj et al, (2016)[2] (global level)
- Van Soest et al, (2017)[3] global level)
- VanDyck et al (2016)[4] (global and country level)
- Kuramochi et al, (2016)[5] (country level)

The results are shown in Supplementary Figures 4-7. In general the GHG emission level by 2030 for the *national policies* scenario are somewhat higher compared to those from Rogelj, et al. [2], van Soest, et al. [2] and Vandyck, et al. [4], while GHG emission levels for the 2 °C scenario in line. The result is a larger emissions gap between national policies and emission levels by 2030 consistent with cost-optimal 2°C scenarios. At G20 economy level, this study is similar (especially median estimates) to national policies scenarios from Kuramochi, et al. [5] and Vandyck, et al. [4], except for the EU and USA for which GHG emissions in this study are slightly higher. The large range of emission levels for China representing national policy implementation is consistent with the outcomes from Kuramochi, et al. [5] and Vandyck, et al. [4] that also differ significantly.

4 Supplementary Note: Model documentation and policy implementation

4.1 Integrated Assessment Model descriptions

The model exercise in this paper was done by nine IAMs that have global coverage, and the results for total GHG emissions and final energy were compared with national models that represent one specific G20 economy. Each model implemented the suite of policies discussed in this paper: 1) No new policies, 2) national policies 3) NDC, 4) 2 °C target (carbon budget 1000) and 5) 1.5 °C (carbon budget 400).

The 'National policies' scenario includes implemented policies for G20 countries. The starting point for this scenario is the no-policy scenario, which is based on the SSP2 scenario[20] and describes a middle-of-the-road scenario in terms of economic and population growth and other long-term trends such as technology development. The main drivers of this scenario for the energy and industry sectors are: population, gross domestic product (GDP), lifestyle and technology change from Riahi et al. (2017)[21], Van Vuuren et al. (2017)[22] and for the LULUCF sector: agricultural productivity, bioenergy and wood demand from Fricko et al (2017)[20]. Integrated assessment models can differ in their interpretation of SSP2 storyline concerning GDP growth (three versions).

The policies included in the national policies scenario were selected from a policy database, and resulted in a list of high impact policies. These policies were translated into policy indicators, which is described in the Methods section. How each policy indicator is implemented in each participating integrated assessment model is described in this chapter of the supplementary material. For each integrated assessment model, first a general description of the model structure and main assumptions are given, and second, a general description of climate policy. A more detailed description per policy type (e.g. fuel efficiency standard, emission trading) is provided in Section 2 of this chapter.

IMAGE 3.0 (global)

<u>Model description</u>

IMAGE 3.0 is a comprehensive integrated assessment framework, modelling interacting human and natural systems (Stehfest et. All, 2014) The IMAGE framework is well suited for assessing interactions between human development and the natural environment, including a range of sectors, ecosystems and indicators. The model allows to assess the impacts of human activities on the natural systems and natural resources and how such impacts hamper the provision of ecosystem services to sustain human development. The model framework is suited to a large geographical (usually global) and temporal scale (up to the year 2100).

The IMAGE framework identifies socio-economic pathways, and projects the consequences for energy, land, water and other natural resources, subject to resource availability and quality. Impacts such as air, water and soil emissions, climatic change, and depletion and degradation of remaining stocks (fossil fuels, forests), are calculated and taken into account in future projections. Within the IAM group, different types of models exist, and IMAGE is characterised by relatively detailed biophysical processes and a wide range of environmental indicators.

The IMAGE Energy Regional model (TIMER) has been developed to explore scenarios for the energy system in the broader context of the IMAGE framework. Similar to other IMAGE components, TIMER is a simulation model. The results obtained depend on a single set of deterministic algorithms, according to which the system state in any future year is derived entirely from previous system states. TIMER includes 12 primary energy carriers in 26 world regions and is used to simulate long-

term trends in energy use, issues related to depletion, energy-related greenhouse gas and other air polluting emissions, together with land-use demand for energy crops. The focus is on dynamic relationships in the energy system, such as inertia and learning-by-doing in capital stocks, depletion of the resource base and trade between regions.

Policy implementation

Population and GPD (Dellink et al, 2017) projections from the SSP2 scenario are exogenous input to the model (Van Vuuren et al, 2017) and do not change in the National policies scenario.

The IMAGE 3.0 model consists of several components, of which the TIMER energy model analyses long-term trends in energy demand and supply in the context of the sustainable development challenges (Van Vuuren et al, 2017). Another component enables the long-term trends for agriculture and land use. The carbon tax is the main policy instrument in the TIMER model, but also regulations or (implicit) policy targets can be imposed by changing model input parameters. Policy targets that cover multiple sectors (e.g. intensity targets and renewable shares of final energy) cannot be directly implemented into the model, and are checked the implementation of other policies. If these multiple-sector targets are not met, sector carbon taxes or regulations are imposed iteratively.

A carbon tax is imposed at region or sector (energy supply, industry, transport, buildings) level, and with small model adjustments also specific sub-sectors can be targeted (e.g. F-gas emission reduction targets). An increase in the carbon tax increases the costs of fossil energy carriers relative to the baseline. This induces a response of the energy system and results in an increased allocation of investments into different non-fossil energy technologies. This allocation is calculated by a multinomial logit function that accounts for relative differences in costs and preferences (technologies with lower costs gain larger market shares). It is also possible to impose other taxes or subsidies on for example fossil fuels (e.g. oil tax) or cars (e.g. subsidy for electric cars) that also change the relative costs of technologies.

Regulations (e.g. standards) are implemented into the model by changing input parameters (e.g. car efficiencies, building insulation rate) or by enforcing larger allocation of investments (e.g. in renewable electricity) than calculated by the multinomial logit function in the baseline. For example, car efficiencies and corresponding costs are model input parameters that can be changed to enforce fuel efficiency standards, and minimal renewable electricity targets are input parameters to the model and result in larger proliferation of renewables compared to baseline. This is also used for those policy instruments that were translated into policy indicators (see CD-LINKS protocol), such as feed-in-tariffs that were translated to renewable electricity targets.

Dellink, R., Chateau, J., Lanzi, E. & Magné, B. Long-term economic growth projections in the Shared Socioeconomic Pathways. *Global Environmental Change* **42**, 200-214, doi:10.1016/j.gloenvcha.2015.06.004 (2017).

Stehfest, E., Van Vuuren, D.P., Bouwman, L., Kram, T., Alkemade, R., Bakkenens, M., Biemans, H., Bouwman, A., Den Elzen, M., Janse, J., Lucas, P., Van Minnen, J., Müller, C., Prins, A., (2014) Integrated Assessment of Global Environmental Change with Model description and policy applications IMAGE 3.0. PBL Netherlands Environmental Assessment Agency, Bilthoven.

Van Soest, H.L., de Boer, H.S., Roelfsema, M., den Elzen, M.G.J., Admiraal, A., van Vuuren, D.P., Hof, A.F., van den Berg, M., Harmsen, M.J.H.M., Gernaat, D.E.H.J., Forsell, N. (2017) Early action on Paris Agreement allows for more time to change energy systems. Climatic Change 144, 165-179.

Van Vuuren, D.P., Stehfest, E., Gernaat, D.E.H.J., Doelman, J.C., van den Berg, M., Harmsen, M., de Boer, H.S., Bouwman, L.F., Daioglou, V., Edelenbosch, O.Y., Girod, B., Kram, T., Lassaletta, L., Lucas, P.L., van Meijl, H., Müller, C., van Ruijven, B.J., van der Sluis, S., Tabeau, A. (2017) Energy, land-use and greenhouse gas emissions trajectories under a green growth paradigm. Global Environmental Change 42, 237-250.

MESSAGEix-GLOBIOM_1.0 (global)

Model description

MESSAGE*ix*-GLOBIOM 1.0 integrates the energy engineering model MESSAGE with the land-use model GLOBIOM via soft-linkage into a global integrated assessment modelling framework (Fricko et al., 2017; Krey et al., 2016). It utilizes the ix platform for integrated and cross-sectoral modelling (Huppmann et al., in preparation).

MESSAGE (Model for Energy Supply Strategy Alternatives and their General Environmental Impact) is a linear programming (LP) energy engineering model with global coverage (Riahi et al., 2012; Riahi, Grübler, & Nakicenovic, 2007). As a systems engineering optimization model, MESSAGE is primarily used for medium- to long-term energy system planning, energy policy analysis, and scenario development. The model provides a framework for representing an energy system with all its interdependencies from resource extraction, imports and exports, conversion, transport, and distribution, to the provision of energy end-use services such as light, space conditioning, industrial production processes, and transportation. MESSAGE-Access (Cameron et al., 2016) is a standalone residential cooking energy choice and demand model that can be applied jointly with MESSAGE*ix*-GLOBIOM to estimate implications of energy and climate policies on access to clean cooking fuels. To assess economic implications and to capture economic feedbacks of climate and energy policies, MESSAGE is linked to the aggregated macro-economic model MACRO (Messner & Schrattenholzer, 2000).

Land-use dynamics are modelled with the GLOBIOM (GLobal BIOsphere Management) model, which is a partial-equilibrium model (Havlík et al., 2011; P. Havlík et al., 2014). GLOBIOM represents the competition between different land-use based activities. It includes a detailed representation of the agricultural, forestry and bio-energy sector, which allows for the inclusion of detailed grid-cell information on biophysical constraints and technological costs, as well as a rich set of environmental parameters, incl. comprehensive AFOLU (agriculture, forestry and other land use) GHG emission accounts and irrigation water use. For spatially explicit projections of the change in afforestation, deforestation, forest management, and their related CO_2 emissions, GLOBIOM is coupled with the G4M (Global FORest Model) model (Gusti, 2010; Kindermann, Obersteiner, Rametsteiner, & McCallum, 2006). As outputs, G4M provides estimates of forest area change, carbon uptake and release by forests, and supply of biomass for bioenergy and timber.

MESSAGE*ix*-GLOBIOM covers all greenhouse gas (GHG)-emitting sectors, including energy, industrial processes as well as agriculture and forestry. The emissions of the full basket of greenhouse gases including CO2, CH4, N2O and F-gases (CF4, C2F6, HFC125, HFC134a, HFC143a, HFC227ea, HFC245ca and SF6) as well as other radiatively active substances, such as NOx, volatile organic compounds (VOCs), CO, SO2, and BC/OC is represented in the model. Air pollution implications of the energy system are accounted for in MESSAGE*ix*-GLOBIOM by a linkage to the GAINS (Greenhouse gas and Air pollution INteractions and Synergies) model (Amann et al., 2011). MESSAGE*ix*-GLOBIOM is used in conjunction with MAGICC (Model for Greenhouse gas Induced Climate Change) version 6.8 (Meinshausen, Raper, & Wigley, 2011) for calculating atmospheric concentrations, radiative forcing, and annual-mean global surface air temperature increase.

Policy implementation

The starting point for the national policy scenarios are the MESSAGE-GLOBIOM implementations of the Shared Socio-economic Pathways (Riahi et al., 2017; Fricko et al., 2017)). The (no-policy) baseline scenario, which does not include any policies, is calibrated up until 2010 and is used as a basis for implementing policies, which do not provide specific values for given target years or change relative to a historical base year, but are expressed relative to a business-as-usual development. The scenarios assume GDP and population developments based on the respective SSP storyline, of which SSP2 (Fricko et al., 2017) serves as the central case, describing a middle of the road scenario and provides a mean challenge to climate mitigation and impact within the SSP framework (Riahi et al., 2017).

National policies are implemented in either 2020 or 2030, as the model has ten-year time resolution, at the model region resolution, meaning that national policies are recalculated to corresponding regional targets based on national historic data (Rogelj et al., 2017).

The main policy types implemented cover i.) emission reduction targets, ii.) share targets, iii.) capacity targets as well as iv.) efficiency increase targets. The first three policy types are implemented by directly adding constraints to the model per region. *Emission reduction targets* are derived by combining baseline regional emission levels, which are downscaled to national emission levels (van Vuuren et al., 2007) for countries within a region that do not have specific emission reduction targets, with national emission reduction targets. The derived national emission levels are aggregated back to the model region level, which are implemented as upper constraints on GHG emission levels in the respective time-periods. In order to avoid any rebound effects, in case regions contain countries without emission reduction targets, emission levels for that region are restricted to baseline levels. *Share targets* come in many different variations (e.g., renewable energy share of primary energy, biofuel share in transport). Baseline energy levels are proportionally downscaled to the country level using historical (2010) energy data. These are then used to recalculate the national share targets at the regional level. If within a region, countries have defined different types of share targets, then these are harmonized to the share constraint type used by the largest country, in terms of energy share, within that region, so that the aggregate effect of national targets is modelled within each region. *Capacity targets* defined by countries are recalculated into a constraint requiring relevant production technologies to provide a minimum energy output equivalent to the installed capacity, using regionally specific technology parameters (e.g., conversion efficiency, capacity factor). The fourth type of policy, *efficiency improvement targets*, are implemented via adjusting the autonomous energy efficiency improvement (AEEI) indicators of the MACRO model (linked to MESSAGE) based on the total final energy savings as estimated by the IMAGE model.

Amann, M., Bertok, I., Borken-Kleefeld, J., Cofala, J., Heyes, C., Höglund-Isaksson, L., . . . Winiwarter, W. (2011). Cost-effective control of air quality and greenhouse gases in Europe: modeling and policy applications. *Environ. Model. Softw., 26*, 1489–1501.

Cameron, C., Pachauri, S., Rao, N., McCollum, D., Rogelj, J., & Riahi, K. (2016). Policy trade-offs between climate mitigation and clean cook-stove access in South Asia. *Nature Energy, 1*.

Fricko, O., Havlik, P., Rogelj, J., Klimont, Z., Gusti, M., Johnson, N., . . . Riahi, K. (2017). The marker quantification of the Shared Socioeconomic Pathway 2: A middle-of-the-road scenario for the 21st century. *Global Environmental Change, 42*, 251-267. doi:http://dx.doi.org/10.1016/j.gloenvcha.2016.06.004

Gusti, M. (2010). An algorithm for simulation of forest management decisions in the global forest model. *Штучний інтелект*.

Havlík, P., Schneider, U. A., Schmid, E., Böttcher, H., Fritz, S., Skalský, R., . . . Obersteiner, M. (2011). Global land-use implications of first and second generation biofuel targets. *Energy Policy, 39*(10), 5690 - 5702.

Havlík, P., Valin, H., Herrero, M., Obersteiner, M., Schmid, E., Rufino, M. C., . . . Notenbaert, A. (2014). Climate change mitigation through livestock system transitions. *Proceedings of the National*

Academy of Sciences of the United States of America, 111(10), 3709-3714. doi:10.1073/pnas.1308044111

Huppmann, D., Gidden, M., Fricko, O., Kolp, P., Orthofer, C., Pimmer, M., . . . Krey, V. (in preparation). The MESSAGEix Integrated Assessment Model and the ix modeling platform.

Kindermann, G., Obersteiner, M., Rametsteiner, E., & McCallum, I. (2006). Predicting the deforestation-trend under different carbon-prices. *Carbon Balance and Management, 1*(1), 15.

Krey, V., Havlik, P., Fricko, O., Zilliacus, J., Gidden, M., Strubegger, M., . . . Riahi, K. (2016). *MESSAGE-GLOBIOM 1.0 Documentation*. Retrieved from Laxenburg, Austria: http://data.ene.iiasa.ac.at/message-globiom/

Meinshausen, M., Raper, S. C. B., & Wigley, T. M. L. (2011). Emulating coupled atmosphere-ocean and carbon cycle models with a simpler model, MAGICC6 - Part 1: Model description and calibration. *Atmospheric Chemistry and Physics, 11*(4), 1417-1456. doi:10.5194/acp-11-1417-2011

Messner, S., & Schrattenholzer, L. (2000). MESSAGE-MACRO: linking an energy supply model with a macroeconomic module and solving it iteratively. *Energy, 25*(3), 267-282.

Riahi, K., Dentener, F., Gielen, D., Grubler, A., Jewell, J., Klimont, Z., . . . Wilson, C. (2012). Energy Pathways for Sustainable Development. In *The Global Energy Assessment: Toward a More Sustainable Future.*: IIASA, Laxenburg, Austria and Cambridge University Press, Cambridge, UK.

Riahi, K., Grübler, A., & Nakicenovic, N. (2007). Scenarios of long-term socio-economic and environmental development under climate stabilization. *Technological Forecasting and Social Change, 74*(7), 887-935. doi:10.1016/j.techfore.2006.05.026

Riahi, K. *et al.* The Shared Socioeconomic Pathways and their energy, land use, and greenhouse gas emissions implications: An overview. *Global Environmental Change* **42**, 153-168, doi:10.1016/j.gloenvcha.2016.05.009 (2017).

Rogelj, J. *et al.* Understanding the origin of Paris Agreement emission uncertainties. *Nature Communications* **8**, 15748, doi:10.1038/ncomms15748 (2017).

van Vuuren, D. P., Lucas, P. L. & Hilderink, H. Downscaling drivers of global environmental change: Enabling use of global SRES scenarios at the national and grid levels. *Global Environmental Change* **17**, 114-130, doi:https://doi.org/10.1016/j.gloenvcha.2006.04.004 (2007).

POLES CDL (global)

<u>Model description</u>

The POLES (Prospective Outlook on Long-term Energy Systems) model (Keramidas et al, 2017) is a global partial equilibrium simulation model of the energy sector with an annual step, covering 38 regions world-wide (G20, OECD, principal energy consumers) plus the EU. The model covers 15 fuel supply branches, 30 technologies in power production, 6 in transformation, 15 final demand sectors and corresponding greenhouse gas emissions. GDP and population are exogenous inputs of the model. The model can provide insights of the evolution of global and local technology developments. The model can assess the market uptake and development of various new and established energy technologies as a function of changing scenario conditions. The global coverage allows an adequate capture of the learning effects that usually occur in global markets (Criqui, 2015). The model represents the adjustments of energy supply and demand to prices, while accounting for delayed reaction. POLES can also assess the global primary energy markets and the related international and regional fuel prices under different scenario assumptions. To this end, it includes a detailed representation of the costs in primary energy supply (in particular oil, gas and coal supply), for both

conventional and unconventional resources. Major countries for the oil, coal and gas markets are represented.

The model can therefore be used to analyse the impacts of energy and climate policies, through the comparison of scenarios concerning possible future developments of world energy consumption and corresponding GHG emissions under different assumed policy frameworks (T. Vandyck , 2016). Policies that can be assessed include: energy efficiency, support to renewables, energy taxation/subsidy, technology push or prohibition, access to energy resources, etc.

Mitigation policies are implemented by introducing carbon prices up to the level where emission reduction targets are met: carbon prices affect the average energy prices, inducing energy efficiency responses on the demand side, and the relative prices of different fuels and technologies, leading to adjustments on both the demand side (e.g. fuel switch) and the supply side (e.g. investments in renewables). Non-CO2 emissions in energy and industry are endogenously modelled with potentials derived from literature (marginal abatement cost curves). Air pollutants are also covered (SO2, NOx, VOCs, CO, BC, OC, PM2.5, PM10, NH3) thanks to a linkage with the specialist GAINS model. Projections for agriculture, LULUCF emissions and food indicators are derived from the GLOBIOM model (dynamic look-up of emissions depending on climate policy and biomass-energy use), calibrated on historical emissions and food demand (from UNFCCC, FAO and EDGAR). A full documentation of POLES is available at http://ec.europa.eu/jrc/poles.

Policy implementation

All POLES scenarios have a common socioeconomic context defined by the population (The Ageing Report, EC 2015; UN Population Division, UN 2015) and economic growth (The Ageing Report, EC 2015; World Economic Outlook, IMF April 2017; CIRCLES, OECD 2014). These inputs are broadly consistent with SSP2. All scenarios share assumptions on discount rates for annual energy investments and certain factors representing lifestyle (e.g. urbanization, dwellings size, mobility evolution); energy taxation and subsidies are constant at their observed historical levels.

All scenarios include adopted energy and climate policies worldwide for 2020. The INDC scenario includes all pledges, including conditional contributions up to 2025 or 2030 (depending on the country). For countries not individually represented, pledges of countries belonging to a region are summed into a pledge representative of that region. Only pledges that can be included in the modelling framework of POLES are considered (for instance, POLES does not explicitly represent an objective in reforestation areas).

Most regulatory measures are included in POLES through several instruments: imposed parameters like fuel standards for vehicles or capacity for nuclear; feed-in tariffs for renewable technologies in the power sector; subsidies in liquid biofuel production costs for renewables in transport; additional energy taxation for energy efficiency objectives; economy-wide carbon value for GHG emissions targets. The carbon value affects the whole economy including agriculture and land use, through average energy prices and the relative prices of different fuels and technologies, inducing fuel switch and energy efficiency responses on the demand side and new technology investments on the supply side (e.g. renewables). Emissions reductions in each sector were achieved depending on the economic attractiveness of mitigation options across sectors.

Non-CO2 emissions in energy and industry are endogenously modelled with marginal abatement cost curves derived from literature (GECS 2002, EPA 2012). Air pollutants are also covered (SO2, NOx, VOCs, CO, BC, OC, PM2.5, PM10, NH3) using emission factors derived from the GAINS model. Projections for land use, LULUCF emissions, agriculture and food indicators are derived from the GLOBIOM model (historical data from UNFCCC, FAO and EDGAR); these parameters are modelled via look-up tables for each country/region, depending on climate policy and biomass-energy use (thus replicating marginal abatement cost curves for LULUCF and agriculture).

Keramidas, K, Kitous, A., Després, J., Schmitz, A., POLES-JRC model documentation. EUR 28728 EN, Publications Office of the European Union, Luxembourg, 2017, ISBN 978-92-79-71801-4, doi:10.2760/225347, JRC107387, 2017

P. Criqui, S. Mima, P. Menanteau, and A. Kitous, 'Mitigation strategies and energy technology learning: An assessment with the POLES model', Technological Forecasting and Social Change, vol. 90, no. Part A, pp. 119–136, Jan. 2015.

T. Vandyck, K. Keramidas, B. Saveyn, A. Kitous, and Z. Vrontisi, 'A global stocktake of the Paris pledges: Implications for energy systems and economy', Global Environmental Change, vol. 41, no. Supplement C, pp. 46–63, Nov. 2016.

Commission, E. The 2015 ageing report: economic and budgetary projections for the 28 EU Member States (2013-2060), http://ec.europa.eu/economy_finance/publications/european_economy/2015/ee3_en.htm. (2015).

UN. World Population Prospects, the 2012 Revision, https://www.un.org/en/development/desa/publications/world-population-prospects-2015-revision.html. (2015).

IMF. World Economic Outlook, https://www.imf.org/en/Publications/WEO/Issues/2017/09/19/world-economic-outlook-october-2017. (2017).

OECD. CIRCLES, https://www.oecd.org/env/indicators-modelling-outlooks/circle.htm. (2014).
US EPA. *Global Anthropogenic Non-CO2 Greenhouse Gas Emissions: 1990-2030: Revised Version 2012*, <http://www.epa.gov/climatechange/Downloads/EPAactivities/EPA_Global_NonCO2_Projections_Dec2012.pdf> (2012).

US EPA. *Global Anthropogenic Non-CO2 Greenhouse Gas Emissions: 1990-2030: Revised Version 2012*, <http://www.epa.gov/climatechange/Downloads/EPAactivities/EPA_Global_NonCO2_Projections_Dec2012.pdf> (2012)

GECS. Greenhouse gas emission control strategies - Final report, https://agritrop.cirad.fr/511091/1/document_511091.pdf. (2002).

REMIND-MAgPIE 1.7-3.0 (global)

Model description

REMIND models the global energy-economy-climate system for 11 world regions and for the time horizon until 2100. For the present study, REMIND in its version 1.7 was used. REMIND represents five individual countries (China, India, Japan, United States of America, and Russia) and six aggregated regions formed by the remaining countries (European Union, Latin America, sub-Saharan Africa without South Africa, Middle East / North Africa / Central Asia, other Asia, Rest of the World). For each region, intertemporal welfare is optimized based on a Ramsey-type macro-economic growth model. The model explicitly represents trade in final goods, primary energy carriers, and in the case of climate policy, emission allowances and computes simultaneous and intertemporal market equilibria based on an iterative procedure. Macro-economic production factors are capital,

labour, and final energy. REMIND uses economic output for investments in the macro-economic capital stock as well as consumption, trade, and energy system expenditures.

By coupling a macroeconomic equilibrium model with a technology-detailed energy model, REMIND combines the major strengths of bottom-up and top-down models. The macro-economic core and the energy system module are hard-linked via the final energy demand and costs incurred by the energy system. A production function with constant elasticity of substitution (nested CES production function) determines the final energy demand. For the baseline scenario, final energy demands pathways are calibrated to regressions of historic demand patterns. More than 50 technologies are available for the conversion of primary energy into secondary energy carriers as well as for the distribution of secondary energy carriers into final energy.

Policy implementation

All scenarios are based on socio-economic assumptions from the SSP2 scenario (Fricko et al. 2017), including GDP, population, demand for energy and food, technology availability and costs, etc.. In all scenarios, fuel taxes and subsidies are represented (Jewell et al. 2018). Taxes are assumed to stay constant, while subsidies are assumed to be phased out until 2050 in all regions and all scenarios.

Implementation of climate policies differs depending on the time horizon and scenario. Until 2020, currently observed policies are implemented as lower bounds for absolute technology deployment for various low-carbon technologies (bio-energy, photovoltaic, wind, nuclear, electric vehicles), or share targets for renewables or low-carbon energy in different countries. Furthermore, to mimic the effect of fuel efficiency standards in transport, upper bounds on final energy usage informed by results from the IMAGE model are implemented.

In the NDC scenario, all the national policies were implemented as well, and extended to 2030 if applicable, but in addition regionally differentiated carbon taxes were implemented. These were iteratively adjusted to secure achieving the economy-wide emission targets from the NDCs for each region. Carbon taxes apply to all greenhouse gas emissions, using 100 year global warming potentials. The interplay between carbon prices and sectoral policies is bidirectional: In some cases, the carbon prices required for reaching the 2030 emission target lead to an overachievement of the policy indicators, and become non-binding. In other cases the technology policies are binding and lead to lower carbon prices than would be required without those additional policies. This is important to keep in mind for the interpretation of the resulting regionally differentiated carbon prices.

In scenarios with long-term global carbon budgets of 400, 1000 and 1600 Gt CO2 from 2011-2100, globally harmonized carbon taxes are iteratively adjusted, such that the budget target is met. The temporal profile of the carbon tax is exogenously set to an exponential increase with 5% per year until 2060, and linear increase thereafter. This profile is chosen so as to limit the temporal overshoot of the carbon budget, and thus reduce the need for removal of CO2 from the atmosphere.

Fricko, O., Havlik, P., Rogelj, J., Klimont, Z., Gusti, M., Johnson, N., . . . Riahi, K. (2017). The marker quantification of the Shared Socioeconomic Pathway 2: A middle-of-the-road scenario for the 21st century. *Global Environmental Change, 42*, 251-267. doi:http://dx.doi.org/10.1016/j.gloenvcha.2016.06.004

Jewell, J. *et al.* Limited emission reductions from fuel subsidy removal except in energy-exporting regions. *Nature* **554**, 229, doi:10.1038/nature25467

https://www.nature.com/articles/nature25467#supplementary-information (2018).

REMIND uses reduced-form emulators derived from the detailed land-use and agricultural model MAgPIE (Lotze-Campen, H. *et al*, 2008; Popp, A. *et al*, 2014) to represent land-use and agricultural emissions as well as bioenergy supply and other land-based mitigation options. Beyond CO_2, REMIND also represents emissions and mitigation options of major non-CO_2 greenhouse gases (Strefler et al., 2014; EPA, 2013).

Lotze-Campen, H. *et al.* Global food demand, productivity growth, and the scarcity of land and water resources: a spatially explicit mathematical programming approach. *Agricultural Economics* **39,** 325–338 (2008)

Popp, A. *et al.* Land-use protection for climate change mitigation. *Nature Clim. Change* **4,** 1095–1098 (2014).

Strefler, J., Luderer, G., Aboumahboub, T. & Kriegler, E. Economic impacts of alternative greenhouse gas emission metrics: a model-based assessment. *Climatic Change* (2014). doi:10.1007/s10584-014-1188-y

EPA. Global Mitigation of Non-CO2 Greenhouse Gases: 2010-2030. EPA–430–R–13–011 (2013).

WITCH2016 (global)

<u>Model description</u>

WITCH-GLOBIOM (World Induced Technical Change Hybrid) is an integrated assessment model designed to assess climate change mitigation and adaptation policies. It is developed and maintained at the Fondazione Eni Enrico Mattei and the Centro Euro-Mediterraneo sui Cambiamenti Climatici. It is a global integrated assessment model with two main distinguishing features: a regional game-theoretic setup, and an endogenous treatment of technological innovation for energy conservation and decarbonisation. A top-down inter-temporal Ramsey-type optimal growth model is hard linked with a representation of the energy sector described in a bottom-up fashion, hence the hybrid denomination. The regional and intertemporal dimensions of the model make it possible to differentiate and assess the optimal response to several climate and energy policies across regions and over time. The non-cooperative nature of international relationships is explicitly accounted for via an iterative algorithm which yields the open-loop Nash equilibrium between the simultaneous activity of a set of representative regions. Regional strategic actions interrelate through GHG emissions, dependence on exhaustible natural resources, trade of fossil fuels and carbon permits, and technological R&D spill overs. R&D investments are directed towards either energy efficiency improvements or development of carbon-free breakthrough technologies. Such innovation cumulates over time and spills across countries in the form of knowledge stocks and flows.

The competition for land use between agriculture, forestry, and bioenergy, which are the main land-based production sectors, is described through a soft link with a land use and forestry model (GLOBIOM, Global Biosphere Management Model, see (Havlík et al., 2014)). A climate model (MAGICC) is used to compute climate variables from GHG emission levels and an air pollution model (FASST) is linked to compute air pollutant concentrations. While for this exercise WITCH is used for cost-effective mitigation analysis, the model supports climate feedback on the economy to determine the optimal adaptation strategy, accounting for both proactive and reactive adaptation expenditures.

WITCH-GLOBIOM represents the world in a set of a varying number of macro regions – for the present study, the eversion with 13 representative native regions has been used; for each, it generates the optimal mitigation strategy for the long-term (from 2005 to 2100) as a response to

external constraints on emissions. A model description is available in (Bosetti et al., 2006), and (Emmerling et al., 2016), and a full documentation can be found at http://doc.witchmodel.org.

Policy implementation

Population (KC and Lutz 2017) and GPD (Dellink et al. 2017) in WITCH model for the SSP2 scenario are exogenous inputs. The model includes a portfolio of policy instruments (Aldy et. Al, 2016) . In this paper we implement the NDCs and national policy emission targets, and also the NDCs and national policy explicit energy targets, such as energy intensity, efficiency, renewable and technology specific deployment targets. Concerning the sector specific policies, the WITCH model can only individualize the transport sector. Policies targeting the transport sector have been implemented at an aggregated sectoral level.

The regional shadow prices of the short term policies, imposed by the emission targets of the NDCs and national policies are used to price all emissions including the land use sector by region. This way the effort imposed on the land use is equal to the one imposed on the energy system.

The long term targets are implemented through a sector-wide carbon tax imposed on the energy and land use systems by region in such way that meets the scenario specifications (Emmerling et al, 2016).

Aldy, J. *et al.* Economic tools to promote transparency and comparability in the Paris Agreement. *Nature Climate Change* **6**, 1000, doi:10.1038/nclimate3106 (2016).

Bosetti, V., Carraro, C., Galeotti, M., Massetti, E., Tavoni, M., 2006. WITCH A World Induced Technical Change Hybrid Model. The Energy Journal 27, 13–37.

Emmerling, J., Drouet, L., Reis, L.A., Bevione, M., Berger, L., Bosetti, V., Carrara, S., Cian, E.D., D'Aertrycke, G.D.M., Longden, T., Malpede, M., Marangoni, G., Sferra, F., Tavoni, M., Witajewski-Baltvilks, J., Havlik, P., 2016. The WITCH 2016 Model - Documentation and Implementation of the Shared Socioeconomic Pathways (Working Paper No. 2016.42). Fondazione Eni Enrico Mattei.

Havlík, P., Valin, H., Herrero, M., Obersteiner, M., Schmid, E., Rufino, M.C., Mosnier, A., Thornton, P.K., Böttcher, H., Conant, R.T., Frank, S., Fritz, S., Fuss, S., Kraxner, F., Notenbaert, A., 2014. Climate change mitigation through livestock system transitions. PNAS 111, 3709–3714. doi:10.1073/pnas.1308044111

Kc, S. & Lutz, W. The human core of the shared socioeconomic pathways: Population scenarios by age, sex and level of education for all countries to 2100. *Global Environmental Change* **42**, 181-192, doi:https://doi.org/10.1016/j.gloenvcha.2014.06.004 (2017).

Dellink, R., Chateau, J., Lanzi, E. & Magné, B. Long-term economic growth projections in the Shared Socioeconomic Pathways. *Global Environmental Change* **42**, 200-214, doi:10.1016/j.gloenvcha.2015.06.004 (2017).

AIM V2.1 (national (Japan)/global)

Model description

AIM V2.1 is a one-year-step recursive-type dynamic general equilibrium model that covers all regions of the world. The AIM/CGE model includes 17 regions and 42 industrial classifications. For appropriate assessment of bioenergy and land use competition, agricultural sectors are also highly disaggregated. Details of the model structure and mathematical formulae are described by Fujimori

et al.. The production sectors are assumed to maximize profits under multi-nested constant elasticity substitution (CES) functions and each input price. Energy transformation sectors input energy and value added are fixed coefficients of output. They are treated in this manner to deal with energy conversion efficiency appropriately in the energy transformation sectors. Power generation values from several energy sources are combined with a Logit function. This functional form was used to ensure energy balance because the CES function does not guarantee an energy balance. Household expenditures on each commodity are described by a linear expenditure system function. The parameters adopted in the linear expenditure system function are recursively updated in accordance with income elasticity assumptions. In addition to energy-related CO_2, CO_2 from other sources, CH_4, N_2O, and fluorinated gases (F-gases) are treated as GHGs in the model. Energy-related emissions are associated with fossil fuel feedstock use. The non-energy-related CO_2 emissions consist of land use change and industrial processes. Land use change emissions are derived from the forest area change relative to the previous year multiplied by the carbon stock density, which is differentiated by AEZs (Global Agro-Ecological Zones). Non-energy-related emissions other than land use change emissions are assumed to be in proportion to the level of each activity (such as output). CH_4 has a range of sources, mainly the rice production, livestock, fossil fuel mining, and waste management sectors. N_2O is emitted as a result of fertilizer application and livestock manure management, and by the chemical industry. F-gases are emitted mainly from refrigerants used in air conditioners and cooling devices in industry. Air pollutant gases (BC, CO, NH_3, NMVOC, NO_X, OC, SO_2) are also associated with fuel combustion and activity levels. Essentially, emissions factors change over time with the implementation of air pollutant removal technologies and relevant legislation.

Policy implementation

All parameter assumptions to quantify the SSP2 scenario is described in (Fuijimori et al, 2017). Population and GDP are exogenous sources.

The carbon tax is the main policy instrument in the AIM model, but also regulations or (implicit) policy indicators can be imposed by changing model input parameters. Some policy targets that either cover multiple sectors or that are not directly linked with the model input parameters (e.g. intensity targets, renewable capacity targets) cannot be directly implemented into the model, and are checked afterwards. If these targets are not met, parameters (e.g. representing energy efficiency) are imposed iteratively.

We basically change the logit function parameters for the share of fuel usages and power generation technological shares. For the energy consumption or energy intensity targets, we have controlled the autonomous energy efficiency improvement parameters iteratively because the energy consumption itself is affected by the price changes which only can be seen after the model simulation. We also implement the fuel taxes by changing the current tax level.

Fujimori S., Hasegawa S., Masui T. (2017) AIM/CGE V2.0: Basic Feature of the Model. In: Fujimori S., Kainuma M., Masui T., Post-2020 Climate Action: Global and Asian Perspective, Springer, 305-328

Fujimori S., Masui T., Matsuoka Y. (2017) AIM/CGE V2.0 Model Formula. In: Fujimori S., Kainuma M., Masui T., Post-2020 Climate Action: Global and Asian Perspective, Springer, 201-303

Fujimori S., Hasegawa T., Masui T., Takahashi K., Herran D.S., Dai H., Hijioka Y., Kainuma M. (2017) SSP3: AIM implementation of Shared Socioeconomic Pathways. Global Environmental Change, 42, 268-283

AIM/Enduse [Japan]

Model description

AIM/Enduse is a partial equilibrium, dynamic recursive model developed by the National Institute for Environmental Studies (NIES), which is characterized by the detailed descriptions of energy technologies in the end-use sectors as well as the energy supply sectors in Japan. This model is characterized by detailed representation of technologies, in which technologies are selected by linear programming minimizing total energy system costs given exogenous parameters such as energy service demands, energy prices, technological parameters, and carbon prices or emissions constraints. It includes non-CO_2 greenhouse gases covered by the Kyoto protocol, and these emissions are converted into CO_2-equivalents using GWP100 factors taken from the IPCC AR4. This model covers not only energy sectors but also non-energy sectors such as industrial processes and waste management, but AFOLU sector is not taken into account. It covers 10 sub-regions in Japan which is broadly coinciding with the areas of 10 public power supply firms, so as to consider characteristics of energy supply and demand across the various-regions. The electricity dispatch module, that is hard-linked with the energy end-use and other energy supply sectors module, explicitly represents the load curve in each region, and capacity of electricity interconnection between sub-regions.

Policy implementation

In AIM/Enduse [Japan], energy technologies are selected based on linear programming and minimize total energy system costs given the exogenous parameters, such as energy service demands, energy prices, technological parameters, and emission prices. The socio-economic conditions, such as population and gross domestic products (GDP), are taken from Nationally Determined Contribution (NDC) scenario in Japan, see Oshiro et al. (2017) for more detail. Even in the no policy scenario, where no climate policy is implemented, some mitigation options are selected due mainly to increase of energy prices, while the technological change in this scenario is relatively moderate.

In the climate policy scenarios, the main driver is economy-wide carbon pricing, while other sectoral policies are also taken into consideration. For example, in the power sector, renewable target mentioned in the NDC is considered which is to increase its share by 22-24% in 2030. Also, the availability of nuclear power would be largely affected by the political condition rather than technological one, maximum capacity of nuclear power is imposed according to nuclear target mentioned in the NDC. In the energy demand sectors, some sector-specific policies associated with the NDC, such as the building energy standards and the fuel economy standards which have been already implemented or planned, are also taken into account.

Oshiro, K., & Masui, T. (2015). Diffusion of low emission vehicles and their impact on CO2 emission reduction in Japan. Energy Policy, 81, 215-225. doi:10.1016/j.enpol.2014.09.010

Oshiro, K., Kainuma, M., & Masui, T. (2017). Implications of Japan's 2030 target for long-term low emission pathways. Energy Policy, 110, 581-587. doi:10.1016/j.enpol.2017.09.003

Kainuma, M., Matsuoka, Y., & Morita, T. (2003). Climate policy assessment: Asia-Pacific integrated modeling (M. Kainuma, Y. Matsuoka, & T. Morita Eds.). Japan: Springer.

COPPE-COFFEE 1.0 (global) and BLUES (national)

Model description

COPPE-COFFEE model is a global optimization model of the energy and land systems based on the MESSAGE platform. It is an intertemporal optimization model, in which the optimal solution provides the minimum cost of the global energy and land-use systems. The COFFEE (COmputable Framework For Energy and the Environment) model has been developed at COPPE, Brazil, for assessing climate, land, energy and environmental policies, providing relevant information to experts and decision-makers on the possible development strategies and repercussions of long term climate scenarios (Rochedo, 2016).

The model has 18 regions, for which all energy and land systems are modelled from 2010 to 2100, and has detailed estimations for the most relevant greenhouse gases (CO_2, CH_4 and N_2O), including a very detailed set of mitigation options for all sectors. The model is based on exogenous demands for energy services (from all economic sectors) and food products, for all regions.

The energy system model is based on a very detailed representation of energy resources and conversion technologies, including power plants, oil refineries, advanced biofuels, CCS infrastructure, transportation technologies, industrials processes and others (Rochedo, 2016). As for the land system representation, COFFEE presents a singular perspective: the model is completely integrated via hard-link with the energy system, which allows for assessing trade-offs and synergies for mitigation strategies in both the energy and land system. However, the COFFEE model is not spatially explicit, which results in a simplified structure for representation land-use dynamics. COFFEE methodological approach is based on different types of land covers that can be modified between one another. In addition, all land covers are desegregated in categories based in the relative cost of opportunity for agricultural production. Therefore, certain type of land covers can be used for agricultural production, to meet the demand for food (crops, livestock, processed food) and bioenergy (Rochedo, 2016).

The Brazilian Land Use and Energy System (BLUES) model, is a perfect foresight, partial equilibrium model covering the Brazilian energy, industry, buildings, transportation and AFOLU sectors. BLUES divides the country into 5 distinct geographic sub-regions plus a sixth national region for interconnection with the rest of the world through import/export. BLUES is the product of gradual implementation of several versions of a MESSAGE model for Brazil (Borba et al., 2012; Koberle et al., 2015; Nogueira et al., 2014; Rochedo, 2016). It chooses the energy system configuration with the least total system cost over the entire time horizon of the study, in this case 2010 to 2050. The model minimizes costs of the entire energy system, including electricity generation, agriculture, industry, transport and the buildings sectors. BLUES finds optimized mixes for the energy system as a whole, rather than evaluating sectorial optimal solutions. It includes CO2, CH4 and N2O emissions associated with land use, agriculture and livestock, fugitive emissions, fuel combustion, industrial processes and waste treatment.

BLUES has six native regions. One main overarching region into which five sub-regions are nested following the geopolitical division of the country. The energy system is represented in detail across sectors, with over 1500 technologies available in and customized for each of its six native regions. The representation of the land-use system includes forests, savannas, low- and high-capacity pastures, integrated systems, cropland, double cropping, planted forests, and protected areas. Cropland is made up of Land use is also regionalized and customized for each sub-region, with yields and costs varying from region to region. Demand is exogenous but endogenous energy efficiency measures permit demand responses through technological options.

http://themasites.pbl.nl/models/advance/index.php/Model_Documentation_-_BLUES.

Policy implementation

COFFEE model uses SSP2 projections for GDP and population (Dellink, 2017). The storyline of the SSP2 scenario affects the model assumptions, such as those used for determining the energy service and food-related demand for all sectors. More detailed analysis is available in (Rochedo, 2016).

Climate policy is implemented via an emissions budget for CO2. For non-CO2 GHGs, an emissions price is implemented, which is in line the globally determined carbon price (interactively), and then multiplied by the AR4 GWP values for each gas (at this time, only CH4 and N2O).

Generally, regulations and policies are implemented in the model via constraints as part of the linear programming algorithms. Specifically, we differ between absolute regulations (e.g. renewable capacity target) and relative regulations (e.g. share of renewables, share of electric vehicles). The former is modelled by minimum capacity/activity constraints, whilst the latter are modelled with user-defined constraint that represent the share equation. Other policies, such as efficiency standards, are modelled by limiting the available set of technologies in the model. For instance, efficiency standard of power plants is modelled by limiting the expansion of lower efficiency power plants, favouring new plants with higher efficiency and/or lower emission factor. The same approach is used for implementing efficiency standard in the transportation sector.

Policy targets that cover multiple sectors or are set as an intensity of GDP or population (e.g. intensity targets, renewable shares of final energy) are very difficult to be directly implemented into the model. The results are analysed and, if the targets are not met, modification to the constrains and carbon cost are adjusted iteratively.

The BLUES model uses SSP2 GDP projections (Dellink et al., 2017) as a starting point, and then adjust them to match historical rates and short-term growth projections by the Brazilian Central Bank (BCB, 2015). The middle SSP2 scenario has estimates for Brazilian GDP annual growth rates averaging 2.2% annual average GDP growth rate for the 2010-2050 period (SSP database, 2015; Riahi et al, 2017). Although such sustained growth rates might have been reasonable to expect a few years ago, recent developments caused a marked reduction in economic activity in Brazil that has made such estimates obsolete. The average growth rate for the period 2011-2014 was just 1.5% per year (ADVFN, 2015; IGBE, 2015). The most recent estimates published by the Brazilian Central Bank indicate Brazilian GDP shrinking by 3.81% in 2015, shrinking again by 3.54% in 2016, and returning to modest growth in subsequent years (BCB, 2018).

In order to create realistic GDP projections for Brazil, we adjust SSP2 growth rates by replacing average growth rates for the periods 2010-2015 and 2015-2020 by average historic and projected rates derived from BCB, 2018). The resulting projection is shown in Table 1, which translates to an annual average of 1.9% for the whole period 2010-2050 (Koberle, 2018). This 1.9% annual growth rate compounds over 40 years, resulting in a Brazilian GDP in 2050 that more than doubles compared to 2010.

GDP projections for Brazil in the BLUES model framework Source: (Köberle, 2018)

2010-2015; 1.5%;

2015-2020; 0.3%;

2020-2025; 2.8%;

2025-2030; 2.4%;

2030-2035; 2.3%;

2035-2040; 2.1%;

2040-2045; 1.9%;

2045-2050: 1.8%

Regulation (e.g energy targets) are implemented in the model as follows via constraints on capacity and/or activity of specific technologies. For example, car efficiencies and corresponding costs are model input parameters that can be changed to enforce fuel efficiency standards, or minimal renewable electricity targets are input parameters to the model and result in higher proliferation of renewables compared to baseline. This is also used for those policy instruments that were translated into policy targets (see CD-LINKS protocol), such as feed-in-tariffs that were translated to renewable electricity targets. Net-zero deforestation is imposed after 2030 to reflect the required afforestation of more than 30 Mha, to compensate for private land currently with a deficit of the legal requirement for natural vegetation stipulated by the 2012 Forest Code (Soares-filho et al., 2014). NDC policies for land use are implemented via constraints imposing the target area for each land use measure via the areas established in the Brazilian NDC (GofB, 2015) and via the Low Carbon Agriculture Plan, or Plano ABC (MAPA, 2012)

Climate policy is implemented via an emissions budget for CO2 and, for non-CO2 GHGs, an emissions price is implemented in line with a globally determined carbon price from global model runs multiplied by the AR4 GWP values for each gas.

ADVFN, 2015. PIB [WWW Document]. Indicadores Econ.

BCB, 2018. Séries de estatísticas consolidadas. Sist. Expect. Merc.

BCB, 2015. Sistema de Expectativas de Mercado [WWW Document]. Banco Cent. do Bras. Séries estatísticas consolidadas. URL https://www3.bcb.gov.br/expectativas/publico/consulta/serieestatisticas (accessed 9.22.15).

Dellink, R., Chateau, J., Lanzi, E., Magné, B., 2017. Long-term economic growth projections in the Shared Socioeconomic Pathways. Glob. Environ. Chang. 42, 200–214. doi:10.1016/j.gloenvcha.2015.06.004

GofB, 2015. Intended Nationally Determined Contribution (INDC) towards achieving the objective of the UNFCCC. Brasilia, Brasil: Presidencia da Republica.

IBGE, 2015. Contas Nacionais Trimestrais [WWW Document]. Sist. Contas Nac. Trimest. URL http://www.ibge.gov.br/home/estatistica/indicadores/pib/defaultcnt.shtm (accessed 10.2.15).

Köberle, A., 2018. Implementation of Land Use in an Energy System Model to Study the Long-Term Impacts of Bioenergy in Brazil and its Sensitivity to the Choice of Agricultural Greenhouse Gas Emission Factors. Federal University of Rio de Janeiro.

MAPA, 2012. Plano Setorial de Mitigação e Adaptação às Mudanças Climáticas para Consolidação da Economia de Baixa Emissão de Carbono na Agricultura – PLANO ABC. Brasilia: Ministério da Agricultura, Pecuária e Abastecimento (MAPA).

Riahi, K. *et al.* The Shared Socioeconomic Pathways and their energy, land use, and greenhouse gas emissions implications: An overview. *Global Environmental Change* **42**, 153-168, doi:10.1016/j.gloenvcha.2016.05.009 (2017).

Rochedo, P., 2016. Development of a global integrated energy model to evaluate the Brazilian role in climate change mitigation scenario. Federal University of Rio de Janeiro.

Soares-filho, B., Rajão, R., Macedo, M., Carneiro, A., Costa, W., Coe, M., Rodrigues, H., Alencar, A., 2014. Cracking Brazil's Forest Code. Science (80-.). 344, 363–364.

SSP database. *SSP Database (Shared Socioeconomic Pathways) - Version 1.0, https://secure.iiasa.ac.at/web-apps/ene/SspDb*, <https://secure.iiasa.ac.at/web-apps/ene/SspDb> (2015).

DNE21+ V.14 (national (Japan)/global)

Model description

Dynamic New Earth 21 Plus (DNE21+) is an energy and global warming mitigation assessment model developed by the Research Institute of Innovative Technologies for the Earth (RITE) (Akimoto et al., 2010; Akimoto, 2014; RITE, 2015). The model is an intertemporal linear programming model for assessment of global energy systems and global warming mitigation in which the worldwide costs are to be minimized. The model represents regional differences, and assesses detailed energy-related CO2 emission reduction technologies up to 2050.When any emission restriction (for example, an upper limit of emissions, emission reduction targets, targets of energy or emission intensity improvements, or carbon taxes) is applied, the model specifies the energy systems whose costs are minimized, meeting all the assumed requirements, including assumed production for industries such as iron and steel, cement, and paper and pulp, transportation by automobile, bus, and truck, and other energy demands. The energy supply sectors are hard-linked with the energy end-use sectors, including energy exporting/importing, and the lifetimes of facilities are taken into account so that assessments are made with complete consistency kept over the energy systems. Salient features of the model include: analysis of regional differences between 54 world regions while maintaining common assumptions and interrelationships; a detailed evaluation of global warming response measures that involves modelling of about 300 specific technologies that help suppress global warming; and explicit facility replacement considerations over the entire time period. The model assumes energy efficiency improvements of several kinds of technologies and cost reductions of renewable energies, carbon dioxide capture and storage (CCS) and so on for the future within the plausible ranges based on the literature.

Policy implementation

The model is based on the baseline of the SSP2 scenario (Fricko et al, 2017). Near-term policies are implemented by translating CD-LINKS protocol (policy indicators) into additional constraints which are suitable for DNE21+. GHG emissions reduction target, CO2 intensity target, Energy intensity targets for total primary energy supply (TPES) and industry sector, energy consumption reduction in TPES and industry sector relative to baseline, cap on TPES and coal consumption, gas and oil import, share of gas and non-fossil energy in TPES, share of renewables in TPES and electricity, renewable electricity production, electricity capacity of biomass, hydro, solar and wind, share of renewables in transport, biofuel share in transport, number of electric and plug-in vehicles, energy savings and CO2 emissions reduction relative to baseline in building sector are explicitly represented by converting the figures of the targets into input parameters of the model. For power plant standards and transport fuel efficiency standards, specific technology options by region by time point, e.g., low-efficiency coal power plant or small low-efficiency internal combustion engine passenger vehicle, which does not meet the standards are excluded in the model.

In terms of long-term CO2 budget, global CO2 budget is exposed as a constraint for policy scenarios.

Akimoto, K., Sano, F., Homma, T., Oda, J., Nagashima, M., Kii, M., Estimates of GHG emission reduction potential by country, sector, and cost, Energy Policy, Vol. 38 pp 3384-3393, 2010.

Akimoto, K.., Sano, F., Homma, T., Tokushige, K., Nagashima, M., Tomoda, M., Assessment of the emission reduction target of halving CO2 emissions by 2050: Macro-factors analysis and model

analysis under newly developed socio-economic scenarios, Energy Strategy Reviews, Vol. 2, pp 246-256, 2014.

Fricko, O. *et al.* The marker quantification of the Shared Socioeconomic Pathway 2: A middle-of-the-road scenario for the 21st century. *Global Environmental Change* **42**, 251-267, doi:https://doi.org/10.1016/j.gloenvcha.2016.06.004 (2017).

RITE, RITE GHG Mitigation Assessment Model DNE21+, 2015.

http://www.rite.or.jp/Japanese/labo/sysken/about-global-warming/download-data/RITE_GHGMitigationAssessmentModel_20150130.pdf

GEM-E3 (global)

Model description

GEM-E3 model is a hybrid, recursive dynamic general equilibrium model that features a highly detailed regional and sectoral representation (Capros, 2014; E3MLab, 2017). The model provides insights on the macroeconomic and sectoral impacts of the interactions of the environment, the economy and the energy system. GEM-E3 allows for a consistent comparative analysis of policy scenarios, ensuring that in all scenarios, the economic system remains in general equilibrium. The model has been calibrated to the latest statistics (GTAP 9, IEA, UN, ILO) while Eurostat statistics have been included instead of the GTAP IO tables for the EU Member States. The GEM-E3 model simultaneously calculates the equilibrium in goods and service markets, as well as in the labour and capital markets based on an optimization of objective functions (welfare for households and cost for firms), and includes projections of: full Input-Output tables by country/region, national accounts, employment, balance of payments, public finance and revenues, household consumption, energy use and supply, GHG emissions and atmospheric pollutants. The model is modularly built allowing the user to select among a number of alternative closure options and market institutional regimes depending on the issue under study. Production functions feature a CES structure and include capital, labour, energy and intermediate goods, while the formulation of production technologies happens in an endogenous manner allowing for price-driven derivation of all intermediate consumption and the services from capital and labour. The model simulates consumer behaviour and explicitly differentiates durable and disposable goods and services. The simulation framework is dynamic, recursive over time, linked in time though the accumulation of capital and equipment. The GEM-E3 regions are linked via endogenous bilateral trade in line with the Armington assumption. This model version features 19 countries/regions, explicitly representing the G-20 members apart from those that are Members of the European Union, as EU28 is represented as one region. The sectoral detail of this model version is high, with 39 separate economic activities, including a distinct representation of the sectors that manufacture low-carbon power supply technologies, electric cars and advanced appliances. In addition, the model includes a detailed representation of the power generation system (10 power technologies) and a highly detailed transport supply module (private and public transport modes). Key novel features of the GEM-E3 model include the involuntary unemployment and an explicit representation of the financial sector. In addition, the GEM-E3 environmental module covers all GHG emissions and a wide range of abatement options, as well as a thoroughly designed carbon market structure (e.g. grandfathering, auctioning, alternative recycling mechanisms) providing flexibility instruments that allow for a variety of options of emission abatement policies.

Policy implementation

The National Policies scenario (Reference scenario) develops on exogenous assumptions on main socio-economic drivers. The GDP growth assumptions are in line with projections by DG ECFIN, OECD, IMF and World Bank for the short-term to 2020 and then develop in line with the SSP2 scenario

projections. Population growth (UN), labor market projections (ILO) and emissions (SSP2) are also considered during the construction of the National Policies scenario, along with the development of technological growth and the respective productivities.

The GEM-E3 model features a detailed representation of the power generation system (10 power technologies) and the transport sector, along with the respective bottom-up power supply and transport modules (private and public transport modes, see Karkatsoulis et al. 2017). Nevertheless, for this application, the GEM-E3 methodological framework does not make use of the bottom-up endogenous, energy system modules for power supply and transport, but instead, features a one-way soft linkage with other energy system or Integrated Assessment models, and in particular with PRIMES EU28 energy system model and IMAGE IAM model for the non-EU regions (see Vrontisi et al, 2019) for more details on the methodological approach). Through this soft-link, an implicit implementation of certain policies is achieved, in accordance with the implementation incorporated by the energy system or IAM model that is used for the soft-link. This approach enables a consistent energy system projection, even in the lack of detailed power technology representation and other relevant limitations.

Karkatsoulis P, Siskos P, Paroussos L, Capros P (2017) Simulating deep CO2 emission reduction in transport in a general equilibrium framework: the GEM-E3T model. Transp Res Part D: Transp Environ 55:343–358. https://doi.org/10.1016/j.trd.2016.11.026

Vrontisi, Z., Fragkiadakis, K., Kannavou, M. Capros, P. Energy system transition and macroeconomic impacts of a European decarbonization action towards a below 2 °C climate stabilization. Climatic Change (2019). https://doi.org/10.1007/s10584-019-02440-7

Capros, P., Paroussos, L., Fragkos, P., Tsani, S., Boitier, B., Wagner, F., Busch, S., Resch, G., Blesl, M., Bollen, J.,(2014) "Description of models and scenarios used to assess European decarbonisation pathways", Energy Strategy Reviews, vol 2, issue 3/4, in press, DOI:10.1016/j.esr.2013.12.008

E3MLab. (2017). GEM-E3 Model Manual 2017. http://www.e3mlab.ntua.gr/e3mlab/GEM%20-%20E3%20Manual/GEM-E3_manual_2017.pdf

P. Karkatsoulis, P. Siskos, L. Paroussos, P. Capros, (2016), Simulating deep CO2 emission reduction in transport in a general equilibrium framework: The GEM-E3T model, Transportation Research, Part D

Pantelis Capros, Leonidas Paroussos, Ioannis Charalampidis, Kostas Fragkiadakis, Panagiotis Karkatsoulis, Stella Tsani, "Assessment of the macroeconomic and sectoral effects of higher electricity and gas prices in the EU: A general equilibrium modeling approach", Energy Strategy Reviews, Volume 9, March 2016, Pages 18-27, ISSN 2211-467X, http://dx.doi.org/10.1016/j.esr.2015.11.002.

***China TIMES (national, China)**

Model description

China TIMES, a dynamic linear programming energy system optimization model, was developed for 5-year intervals extending from 2010 to 2050 on the basis of China MARKAL model (Chen 2005; Chen 2007; Chen 2010). The model incorporates the full range of energy processes including exploitation, conversion, transmission, distribution and end-use (Chen 2014, Chen 2016a; Zhang, 2016; Shi, 2016; Ma 2016; Huang 2017;). Over 500 existing and advanced energy supply and demand technologies are introduced in the model. Five demand sectors, agriculture, industry, commercial, residential (divided into urban and rural) and transportation, are considered and further divided into around 50 sub-sectors (Zhang, 2016; Shi, 2016; Ma 2016). Stock based material flow analysis approach, discrete choices method, Gompertz model and etc. are used to project energy service demands for the 50 sub-sectors according to given social economic development scenarios (Chen 2016b, Yin 2013, Li

2017). Price elasticity for each sub-sector is introduced in the model to allow carbon mitigation to be achieved by change of production mode and consumption pattern and by the deployment of low- and non-carbon technologies. Local air pollutants such as SO2, NOx, PM10, PM2.5 and etc. as well as energy-related water consumption could also be simulated with the China TIMES model

Chen W, 2005. The costs of mitigating carbon emissions in China: findings from China MARKAL-MACRO modelling. Energy Policy, 33(7): 885–896.

Chen W, Wu Z, He J, Gao P, Xu S, 2007. Carbon emission control strategies for China: A comparative study with partial and general equilibrium versions of the China MARKAL model. Energy, 32(1): 59-72.

Chen W, Li H, Wu Z, 2010. Western China energy development and west to east energy transfer: Application of the Western China Sustainable Energy Development Model. Energy Policy, 38(11): 7106–7120.

Chen W, Yin X, Ma D, 2014. A bottom up analysis of China's iron and steel industrial energy consumption and CO2 emissions. Applied Energy, 136:1174-1183.

Chen W, Yin X, Zhang H, 2016a. Towards low carbon development in China: a comparison of national and global models. Climatic Change, 136:95-108.

Chen W, Yin X, Zhang H, and et al., 2016 b. The role of energy service demand in carbon mitigation: combining sector analysis and China TIMES-ED modelling. Informing Energy and Climate Policies Using Energy Systems Models: Insights from Scenario Analysis Increasing the Evidence Base (Giannakidis G., Labriet M., Gallachóir B., Tosato G. eds). Springer.

Ma D, Chen W, Yin X, Wang L, 2016. Quantifying the co-benefits of decarbonisation in China's steel sector: An integrated assessment approach. Applied Energy, 162(C):1225-1237.

Shi J, Chen W, Yin X, 2015. Modelling building's decarbonization with application of China TIMES model. Applied Energy, 162:1303-1312.

Zhang H, Chen W, Huang W, 2016. TIMES modelling of transport sector in China and USA: Comparisons from a decarbonization perspective. Applied Energy, 162:1505-1514.

Huang W, Ma D, Chen W, 2017. Connecting water and energy: Assessing the impacts of carbon and water constraints on China's power sector. Applied Energy, 185:1497-1505

Yin X, Chen W, 2013. Trends and development of steel demand in China: A bottom–up analysis." Resources Policy 38 (4): 407-415.

Li N, Ma D, Chen W, 2017. Quantifying the impacts of decarbonization in China's cement sector: A perspective from an integrated assessment approach. Applied Energy, 185:1840-1848

***IPAC-AIM/technology V1.0 (national, China)**

See http://ipac-model.org.cn/About%20IPAC%20Model.html

***GCAM-USA (national, USA)**

Model description

The Global Change Assessment Model (GCAM) is a partial equilibrium integrated assessment model that couples a suite of dynamic-recursive models of the global energy, economy, agriculture and land-use systems with a reduced-form atmosphere-carbon-cycle-climate model [3]. This study use GCAM-USA, a U.S. focused version of GCAM, that breaks the energy and economy components of the U.S. into 50 states and the District of Colombia in addition to modelling the simultaneous interactions of 31 geopolitical regions outside of the U.S (Iyer et al, 2017a, Iyer et al, 2017b). The principle drivers of GCAM-USA are population growth, labour participation rates and labour productivity, along with representations of resources, technologies and policy. The energy system formulation in GCAM-USA consists of detailed representations of extractions of depletable primary resources such as coal, natural gas, oil and uranium along with renewable sources such as bioenergy, solar, wind and geothermal. Wind, solar and geothermal resources are represented at the state-level for the U.S. and at the level of the 31 other GCAM regions. The supply of bioenergy is modelled in the agriculture and land-use component of the model, along with competition for land among alternative uses, at the national level for the U.S. and at the level of the 31 other GCAM regions. GCAM-USA also includes representations of the processes that transform these resources to final energy carriers which are ultimately used to deliver goods and services demanded by end users in buildings, transportation and industrial sectors. Key energy transformation sectors (refining and electric power), and end-use sectors (buildings, transportation and industry) are modelled at the state-level for the U.S. and at the level of 31 other regions. Each technology in the model has a lifetime, and once an investment is made, technologies operate till the end of their lifetime or are shut down if the variable cost exceeds the market price. The deployment of technologies in GCAM depends on relative costs and is achieved using a logit-choice formulation which is designed to represent decision making among competing options when only some characteristics of the options can be observed.

GCAM Documentation, 2016. http://jgcri.github.io/gcam-doc/toc.html.

Iyer, G., Ledna, C., Clarke, L., McJeon, H., Edmonds, J., Wise, M., 2017a. GCAM-USA Analysis of US Electric Power Sector Transitions. http://www.pnnl.gov/main/publications/external/technical_reports/PNNL-26174.pdf. Pacific Northwest National Laboratory.

Iyer, G., Ledna, C., Clarke, L.E., Edmonds, J., McJeon, H., Kyle, G.P., Williams, J.A., 2017b. Measuring Progress from Nationally Determined Contributions to Mid-Century Strategies. Nature Climate Change (forthcoming).

***AIM-India [IIMA] (national, India)**

Model description

Indian AIM/Enduse is a bottom-up optimization model that provides a techno-economic perspective at national level with sectoral granularity. Built on a disaggregated, sectoral representation of the economy, it provides a detailed characterization of technologies and fuel based on their availability, efficiency levels and costs. It estimates the current and future energy consumption and GHG emissions of all sectors. It uses linear programming to provide a set of technologies that will meet

the exogenous service demand at the least cost while satisfying techno-economic, emissions- and energy-related constraints.

The model has been set up for five major sectors and their respective services, technologies, reference years and discount rates. These sectors are agriculture, industry, power, residential (including commercial) and transportation. Multiple services in each sector have been examined to provide a better understanding of the sector. For example, fifteen industries have been selected to represent the industry sector, while passenger and freight characterize travel demand in the transport sector. The model comprises of over 450 existing, advanced, and futuristic energy supply and demand technologies.

Vishwanathan, S.S., Garg, A., Tiwari, V., Kankal, B., Kapshe, M., Nag, T. (2017) Enhancing Energy Efficiency in India: Assessment of Sectoral Potentials Copenhagen: Copenhagen Centre on Energy Efficiency, UNEP DTU Partnership. ISBN: 978-87-93458-13-0.

Garg, A., Vishwanathan, S.S. and Choksi, P. P. (2017). Informing the international negotiations on climate: Benchmarking of India's national contributions post COP-21. In Garg, H. P., Singh, S. K., and Kandpal, T. C. (Eds.), Advances in Solar Energy Science and Engineering (Volume-4) (pp 355-381) New Delhi: Today & Tomorrow's Printers and Publishers. ISBN: 81-7019-574-4.

Shukla, P. R., Rana, A., Garg, A., Kapshe, M., & Nair, R. (2004). Climate Policy Assessment for India - Applications of Asia-Pacific Integrated Model . Hyderabad: University Press.

Kainuma, M., Matsuka, Y., & Morita, T. (2003). Climate Policy Assessment. Japan: Springer.

***India MARKAL (national, India)**

<u>Model description</u>

TERI's India MARKAL model has been continuously developed over the past two decades and exists as a rich and disaggregated database of energy demand and supply technologies representing India's energy system. The model has been used to develop and examine scenarios to identify and prioritise choices for mitigation and energy efficiency and explore the implications of different emissions constraints. The model has been used to inform policy making within the country (providing inputs for India's NDCs) as well as across a number of national and international studies related with energy security, mitigation and climate change. The model has been used across several studies in the past to analyse implications for India's energy sector. These include Energising India – Towards a Resilient and Equitable Energy System (Bery, S. Mathur R, Ghosh A, 2016), Air Pollutant Emissions Scenario for India, Energy Security Outlook, Pathways to deep decarbonisation and The Energy Report- India 100% Renewable Energy by 2030.

The MARKAL (MARket ALlocation) model is a bottom up dynamic linear programming cost optimization model depicting energy supply, conversion and consumption across demand sectors of a complete generalised energy system. The MARKAL family of models is unique, with applications in a wide variety of settings and global technical support from the international research community. The optimization routine used in the model's solution selects from each of the sources, energy carriers, and transformation technologies to produce the least-cost solution, subject to a variety of constraints. The user defines technology costs, technical characteristics (e.g., conversion efficiencies), and energy service demands.

The current model database, developed by Ritu Mathur, Atul Kumar, Aayushi Awasthy, Sugandha Chauhan, Kabir Sharma, Swapnil Shekhar and Prakriti Prajapati is set up over a 50 year period extending from 2001-2051 at five-yearly intervals originally intended to coincide with the Government of India's Five-Year plans. In the model, the Indian energy sector is disaggregated into five major energy

consuming sectors, namely, agriculture, commercial, industry, residential and transport sectors. End use demands for each of the sectors are derived exogenously using excel based/econometric models.

On the supply side, the model considers the various energy resources that are available both domestically and from abroad for meeting various end-use demands. These include both the conventional energy sources (coal, oil, natural gas, and nuclear) as well as the renewable energy sources (hydro, wind, solar, biomass etc.). The availability of each of these fuels is represented by constraints on the supply side.

The relative energy prices of various forms and source of fuels play an integral role in capturing inter-fuel substitutions within the model. Furthermore, various conversion and process technologies characterized by their respective investment costs, operating and maintenance costs, technical efficiency, life etc. that meet the sectoral end-use demands are also incorporated in the model. In case of technologies that are specific to India, country specific costs are included (capital costs and O&M costs), while globally existing technologies have made use of international sources of data as well. Cost reduction in future in the emerging technologies has also been assumed based on an understanding of the particular technology development.

The database in its current form incorporates 47 end-uses spanning more than 350 technologies. While the demands are set up in line with basic driving parameters such as projected population, urbanization and GDP, the various scenarios include emission constraints and/or reflections of policies and measures that provide varying priorities to alternative energy forms over the modelling timeframe. in order to meet the requirements of CD-LINKS scenarios.

Bery S., Ghosh A. Mathur, R. et al. (2016). Energising India: Towards a Resilient and Equitable Energy System- SAGE Publication, 2016.

***PRIMES_V1 (national, EU)**

Model description

The PRIMES model provides detailed projections of energy demand, supply, prices and investment to the future, covering the entire energy system including emissions for each individual European country and for Europe-wide trade of energy commodities. The distinctive feature of PRIMES is the combination of behavioural modelling following a micro-economic foundation with engineering and system aspects, covering all sectors and markets at a high level of detail. PRIMES focuses on prices as a means of balancing demand and supply simultaneously in several markets for energy and emissions. The model determines market equilibrium volumes by finding the prices of each energy form such that the quantity producers find best to supply matches the quantity consumers wish to use. Investment is generally endogenous in PRIMES and in all sectors, including for purchasing of equipment and vehicles in demand sectors and for building energy producing plants in supply sectors. The model handles dynamics under different anticipation assumptions and projects over a long-term horizon keeping track of technology vintages in all sectors. Technology learning and economies of scale are fully included and are generally endogenous depending on market development. The PRIMES model comprises several sub-models (modules), each one representing the behaviour of a specific (or representative) agent, a demander and/or a supplier of energy. The sub-models link with each other through a model integration algorithm, which determines equilibrium prices in multiple markets and equilibrium volumes meets balancing and overall (e.g. emission) constraints. The agents' behaviours are sector-specific. The modelling draws on structural microeconomics: each demand module formulates a representative agent who maximises benefits (profit, utility, etc.) from energy demand and non-energy inputs (commodities, production factors) subject to prices, budget and other constraints. The constraints relate to activity, comfort, equipment, technology, environment or fuel availability. The supply modules formulate stylised

companies aiming at minimising costs (or maximising profits in model variants focusing on market competition) to meet demand subject to constraints related to capacities, fuel availability, environment, system reliability, etc.

PRIMES is a hybrid model in the sense that it captures technology and engineering detail together with micro and macro interactions and dynamics. Because PRIMES follows a structural modelling approach, in contrast with reduced-form modelling, it integrates technology/engineering details and constraints in economic modelling of behaviours. Microeconomic foundation is a distinguishing feature of the PRIMES model and applies to all sectors. The modelling of decisions draw on economics, but the constraints and possibilities reflect engineering feasibility and restrictions. The model thus combines economics with engineering, ensuring consistency in terms of engineering feasibility, being transparent in terms of system operation and being able to capture features of individual technologies and policies influencing their development. Nevertheless, PRIMES is more aggregated than engineering models, but far more disaggregated than econometric (or reduced form) models. The model performs analytical cost estimations and projections by sector both in demand and supply, as well as for infrastructure. Supply-side modules determine commodity and infrastructure prices by end-use sector (tariffs) by applying various methodologies by sector as appropriate for recovering costs depending on market conditions and regulation where applicable. Pricing and costing include taxes, subsidies, levies and charges, congestion fees, tariffs for use of infrastructure etc. Usually these instruments are exogenous to the model and reflect policy assumptions. The PRIMES model is fully dynamic and has options regarding future anticipation by agents in decision-making. Usually, PRIMES assumes perfect foresight over a short time horizon for demand sectors and perfect foresight over long time horizon for supply sectors. The sub-models solve over the entire projection period in each cycle of interaction between demand and supply and so market equilibrium is dynamic and not static. All formulations of agent behaviours consider technologies, which are either existing at present or expected to become available in the future.

E3MLab, PRIMES Model Version 6 2016-2017 - Detailed model description, (2016). http://www.e3mlab.ntua.gr/e3mlab/PRIMES%20Manual/The%20PRIMES%20MODEL%202016-7.pdf

P. Capros, N. Tasios, M. Kannavou, M. Aslanoglou, C. Delkis E. Kalaintzakis C. Nakos, M. Zampara, S. Evangelopoulou, (2017), "Modelling study contributing to the Impact Assessment of the European Commission of the Electricity Market Design Initiative". https://ec.europa.eu/energy/en/studies

P. Capros, N. Tasios, A. De Vita, L. Mantzos, L. Paroussos. 2012. Model-based analysis of decarbonising the EU economy in the time horizon to 2050, In Energy Strategy Reviews, Volume 1, Issue 2, Pages 76-84, ISSN 2211-467X, https://doi.org/10.1016/j.esr.2012.06.003.

Fragkos, P., Tasios, N., Paroussos, L., Capros, P. & Tsani, S. (2017) "Energy system impacts and policy implications of the European Intended Nationally Determined Contribution and low-carbon pathway to 2050", Energy Policy, Volume 100, January 2017, Pages 216-226

P. Capros, L. Mantzos, L. Parousos, N. Tasios, G. Klaassen, T. Van Ierland. 2011. Analysis of the EU policy package on climate change and renewables, In Energy Policy, Volume 39, Issue 3, 2011, Pages 1476-1485, ISSN 0301-4215, https://doi.org/10.1016/j.enpol.2010.12.020.

P. Capros, N. Tasios, A. De Vita, L. Mantzos, L. Paroussos. 2012. Transformations of the energy system in the context of the decarbonisation of the EU economy in the time horizon to 2050, In Energy Strategy Reviews, Volume 1, Issue 2, Pages 85-96, ISSN 2211-467X, https://doi.org/10.1016/j.esr.2012.06.001.

***RU-TIMES 3.2**

Model description

The RU-TIMES model is based on the Integrated MARKAL-EFOM System aimed at modelling and long-term planning of energy systems and technological processes. The model covers major sectors and industries of Russian economy, including energy industries (power and heat, oil, gas, coal, nuclear, renewables, etc.), ferrous and non-ferrous metallurgy, transport, chemical and petrochemical industry, residential and commercial buildings, foreign trade with energy resources. The current and prospective technology databases are developed for each sector/industry, based on the national statistics and data and international data sources (IEA, OECD, etc.). The technological characteristics, domestic and world primary energy prices, costs of production by different energy sources, GDP growth rate, economic structure, limits of the use of energy sources capacity, as well as some other indicators are used as the exogenous parameters in the model. The scenarios of economic development are developed with regard to the strategic planning documents, such as the Russian energy strategy, long term socio-economic development programs and others. RU-TIMES is a partial equilibrium model, focused on the energy supply and consumption, not covering forestry and land use sectors, agriculture, waste management, and most of non-CO2 gases (includes only CO2 and CH4).

Safonov G et al, Low carbon development strategy in Russia: transition from fossil fuels to green energy sources, Moscow State University - TEIS Publishing House, 2016 [in Russian]

Potashnikov V., PYE S., Exploring national decarbonization pathways and global energy trade flows: a multi-scale analysis//Climate Policy, 2016. – 17 p.

SDSN/IDDRI, Pathways to Deep Decarbonization- 2015 synthesis report, Paris, 2015.

Lightner D., Potashnikov V., Lugovoy O., Low carbon development as a driver for economic growth, Russian Entrepreneurship Journal, Moscow, 2015 [in Russian]

Lugovoy O., Safonov G., Potashnikov V., Gordeev D., Pathways to Deep Decarbonization 2014 report. Russia Chapter, Paris: SDSN/IDDRI, 2014.

Potashnikov V., Lugovoy O., Projections of the energy balance and greenhouse gas emissions based on RU-TIMES model by 2050, Scientific Vestnik of Gaidar's Institute of Economic Policy, #5, Moscow, 2014 [in Russian]

5 Supplementary References

1 Stehfest, E., Van Vuuren, D. P., Bouwman, L. & Kram, T. Integrated Assessment of Global Environmental Change with Model description and policy applications IMAGE 3.0. (PBL Netherlands Environmental Assessment Agency, Bilthoven, 2014).

2 Rogelj, J. *et al.* Paris Agreement climate proposals need a boost to keep warming well below 2 degrees C. *Nature* **534**, 631-639, doi:10.1038/nature18307 (2016).

3 van Soest, H. L. *et al.* Early action on Paris Agreement allows for more time to change energy systems. *Climatic Change* **144**, 165-179, doi:10.1007/s10584-017-2027-8 (2017).

4 Vandyck, T., Keramidas, K., Saveyn, B., Kitous, A. & Vrontisi, Z. A global stocktake of the Paris pledges: Implications for energy systems and economy. *Global Environmental Change* **41**, 46-63, doi:https://doi.org/10.1016/j.gloenvcha.2016.08.006 (2016).

5 Kuramochi, T. *et al.* Greenhouse gas mitigation scenarios for major emitting countries. Analysis of current climate policies and mitigation targets. (New Climate, PBL, IIASA, Cologne, Germany, 2016).

6 den Elzen, M. *et al.* Contribution of the G20 economies to the global impact of the Paris agreement climate proposals. *Climatic Change* **137**, 655-665, doi:10.1007/s10584-016-1700-7 (2016).

7 Grassi, G. & Dentener, F. Quantifying the contribution of the land use sector to the Paris Climate Agreement, https://ec.europa.eu/jrc/en/publication/eur-scientific-and-technical-research-reports/quantifying-contribution-land-use-sector-paris-climate-agreement. (EC Joint Research Centre. Institute for Environment and Sustainability, 2015).

8 UNFCCC. First Biennal report Argentina, https://unfccc.int/resource/docs/natc/argbur1.pdf. (2015).

9 UNFCCC. First Biennal report Indonesia, https://unfccc.int/resource/docs/natc/idnbur1.pdf. (2015).

10 UNFCCC. First and Second Biennal report Turkey, http://unfccc.int/files/national_reports/biennial_reports_and_iar/submitted_biennial_reports/application/pdf/turkey_joint_first_and_second_biennial_report.pdf. (2015).

11 National Government of South Africa. GHG Inventory, http://unfccc.int/resource/docs/natc/zafnir1.pdf. (2015).

12 CD-LINKS. *Protocol for WP3.2 Global low-carbon development pathways, http://www.cd-links.org/wp-content/uploads/2016/06/CD-LINKS-global-exercise-protocol_secondround_for-website.pdf* (2017).

13 CD-LINKS. *High impact policies, http://www.cd-links.org/wp-content/uploads/2016/06/Input-IAM-protocol_CD_LINKS_update_July-2018.xlsx* (2017).

14 Vrontisi, Z. *et al.* Enhancing global climate policy ambition towards a 1.5 °C stabilization: a short-term multi-model assessment. *Environmental Research Letters* **13**, 044039, doi:10.1088/1748-9326/aab53e (2018).

15 Luderer, G. *et al.* Residual fossil CO2 emissions in 1.5–2 °C pathways. *Nature Climate Change* **8**, 626-633, doi:10.1038/s41558-018-0198-6 (2018).

16 Moomaw, W. *et al.* Annex II: Methodology. In IPCC Special Report on Renewable Energy Sources and Climate Change Mitigation. (Intergovernmental Panel on Climate Change, 2011).

17 van Asselt, M. B. A. & Rotmans, J. J. C. C. Uncertainty in Integrated Assessment Modelling. **54**, 75-105, doi:10.1023/a:1015783803445 (2002).

18 Gütschow, J. *et al.* The PRIMAP-hist national historical emissions time series. *Earth Syst. Sci. Data* **8**, 571-603, doi:10.5194/essd-8-571-2016 (2016).

19 Van den Berg et al. Implication of various effort-sharing approaches for regional carbon budgets and emission pathways. *Submitted to Global Environmental Change* (2018).

20 Fricko, O. *et al.* The marker quantification of the Shared Socioeconomic Pathway 2: A middle-of-the-road scenario for the 21st century. *Global Environmental Change* **42**, 251-267, doi:https://doi.org/10.1016/j.gloenvcha.2016.06.004 (2017).
21 Riahi, K. *et al.* The Shared Socioeconomic Pathways and their energy, land use, and greenhouse gas emissions implications: An overview. *Global Environmental Change* **42**, 153-168, doi:10.1016/j.gloenvcha.2016.05.009 (2017).
22 van Vuuren, D. P. *et al.* The Shared Socio-economic Pathways: Trajectories for human development and global environmental change. *Global Environmental Change* **42**, 148-152, doi:10.1016/j.gloenvcha.2016.10.009 (2017).
