# Supplementary material for: Taking stock of national climate policies to evaluate implementation of the Paris Agreement
Source: Nat Commun. 2020 Apr 29;11:2096. doi: 10.1038/s41467-020-15414-6 (PMC7190619; doi:10.1038/s41467-020-15414-6)
Supplement: Supplementary file 3 — Reporting Summary [file 41467_2020_15414_MOESM3_ESM.pdf]

nature research

Corresponding author(s): *Double-blind peer review submissions: write DBPR and your manuscript number here instead of author names.*

Last updated by author(s): *YYYY-MM-DD*

Reporting Summary

Nature Research wishes to improve the reproducibility of the work that we publish. This form provides structure for consistency and transparency in reporting. For further information on Nature Research policies, see Authors & Referees and the Editorial Policy Checklist.

Statistics

For all statistical analyses, confirm that the following items are present in the figure legend, table legend, main text, or Methods section.

n/a	Confirmed	
☒	☐	The exact sample size (n) for each experimental group/condition, given as a discrete number and unit of measurement
☒	☐	A statement on whether measurements were taken from distinct samples or whether the same sample was measured repeatedly
☒	☐	The statistical test(s) used AND whether they are one- or two-sided<br>*Only common tests should be described solely by name; describe more complex techniques in the Methods section.*
☒	☐	A description of all covariates tested
☒	☐	A description of any assumptions or corrections, such as tests of normality and adjustment for multiple comparisons
☒	☐	A full description of the statistical parameters including central tendency (e.g. means) or other basic estimates (e.g. regression coefficient) AND variation (e.g. standard deviation) or associated estimates of uncertainty (e.g. confidence intervals)
☒	☐	For null hypothesis testing, the test statistic (e.g. F, t, r) with confidence intervals, effect sizes, degrees of freedom and P value noted<br>*Give P values as exact values whenever suitable.*
☒	☐	For Bayesian analysis, information on the choice of priors and Markov chain Monte Carlo settings
☒	☐	For hierarchical and complex designs, identification of the appropriate level for tests and full reporting of outcomes
☒	☐	Estimates of effect sizes (e.g. Cohen's d, Pearson's r), indicating how they were calculated

Our web collection on statistics for biologists contains articles on many of the points above.

Software and code

Policy information about availability of computer code

Data collection	The policy database is available at www.policydatabase.org, and derived policy protocol at https://db1.ene.iiasa.ac.at/CDLINKSDB/dsd?Action=htmlpage&page=welcome
Data analysis	Data analysis was done and graphics were produced in R-Studio

For manuscripts utilizing custom algorithms or software that are central to the research but not yet described in published literature, software must be made available to editors/reviewers. We strongly encourage code deposition in a community repository (e.g. GitHub). See the Nature Research guidelines for submitting code & software for further information.

Data

Policy information about availability of data

All manuscripts must include a data availability statement. This statement should provide the following information, where applicable:

- Accession codes, unique identifiers, or web links for publicly available datasets
- A list of figures that have associated raw data
- A description of any restrictions on data availability

The climate, energy and land-use policies that were used in this study were retrieved from the CD-LINKS inventory, which is based on the Climate Policy Database. The scenario protocol and the selection of high-impact policies (Supplementary Data 1) that were included in the protocol are found under Work Package 2 of the deliverables & publications page of the CD-LINKS project. Global data from this article can be found in the open-access CD-LINKS database. Policy relevant data is available in the Global Stocktake tool. Data reported in Figure 1-5 can be found in the Supplementary Data 2.

CD-LINKS inventory http://www.climatepolicydatabase.org/index.php/CDlinks_policy_inventory
Climate policy database http://climatepolicydatabase.org/index.php/Climate_Policy_Database
Deliverables & publications http://www.cd-links.org/?page_id=620

CD-LINKS database https://db1.ene.iiasa.ac.at/CDLINKSDB/dsd?Action=htmlpage&page=30
Global Stocktake tool https://themasites.pbl.nl/global-stocktake-indicators/

Field-specific reporting

Please select the one below that is the best fit for your research. If you are not sure, read the appropriate sections before making your selection.

☐ Life sciences ☐ Behavioural & social sciences ☒ Ecological, evolutionary & environmental sciences

For a reference copy of the document with all sections, see nature.com/documents/nr-reporting-summary-flat.pdf

Ecological, evolutionary & environmental sciences study design

All studies must disclose on these points even when the disclosure is negative.

Study description	Assessment of the global and national impact of current implemented climate policies in G20 countries using nine global integrated assessment models
Research sample	www.climatepolicydatabase.org
Sampling strategy	NA
Data collection	National climate policies were collected by the New Climate Institute and PBL and reviewed by national policy experts
Timing and spatial scale	Collection of policies was done in the year 2015-2017, and for all G20 countries
Data exclusions	A selection from the policy database was made and implemented in the nine integrated assessement models. Around ten policies were selected for each G20 country that were expected to have high impact on greenhouse gas emissions based on literature or national expert opinion, that were adopted by national government trough legislation or executive orders, and no evidence exists of large barriers to implementation.
Reproducibility	Model description and references to documentation were provided, including a detailed description of how policies were implemented in the nine integrated assessment models
Randomization	NA
Blinding	NA

Did the study involve field work? ☐ Yes ☒ No

Reporting for specific materials, systems and methods

We require information from authors about some types of materials, experimental systems and methods used in many studies. Here, indicate whether each material, system or method listed is relevant to your study. If you are not sure if a list item applies to your research, read the appropriate section before selecting a response.

Materials & experimental systems

n/a	Involved in the study
☒	☐ Antibodies
☒	☐ Eukaryotic cell lines
☒	☐ Palaeontology
☒	☐ Animals and other organisms
☒	☐ Human research participants
☒	☐ Clinical data

Methods

n/a	Involved in the study
☒	☐ ChIP-seq
☒	☐ Flow cytometry
☒	☐ MRI-based neuroimaging
